# Mitochondrial-endoplasmic reticulum interactions in lung diseases

**DOI:** 10.1038/s41419-026-09063-8

**Published:** 2026-07-02

**Authors:** Yafang Zhang, Hao Yan, Xianzhi Peng, Lihong Gong, Shenglin Zhang, Dong Liu, Chaoyang Zhang, Cheng Peng, Yunxia Li

**Affiliations:** 1 State Key Laboratory of Southwestern Chinese Medicine Resources, Chengdu, China; 2 School of Pharmacy, Chengdu University of Traditional Chinese Medicine, Chengdu, China; 3 Key Laboratory of Standardization for Chinese Herbal Medicine, Ministry of Education, Chengdu, China

**Keywords:** Apoptosis, Immune cell death

## Abstract

The pathogenesis of lung diseases is highly complex and multifactorial, posing a persistent challenge to global public health, while effective therapeutic options remain limited. Therefore, systematically elucidating the cellular and molecular mechanisms underlying lung injury is a crucial prerequisite for developing novel therapeutic strategies. Accumulating evidence highlights that the endoplasmic reticulum and mitochondria are key organelles driving the progression of lung injury. Notably, mitochondria-associated membranes (MAMs) form a structural and functional bridge between the endoplasmic reticulum and mitochondria, highlighting the essential role of inter-organellar communication in maintaining lung function homeostasis. This review comprehensively summarizes the autonomous mechanisms of the mitochondria and endoplasmic reticulum in lung injury-related diseases, with a further focus on the complex architecture and regulatory mechanisms of MAMs. It emphasizes the molecular mechanisms by which dysfunctional MAMs contribute to the onset and progression of lung injury, including Ca²⁺ signaling, oxidative stress, lipid synthesis and transport, UPR^ER^ and UPR^mt^, mitochondrial homeostasis, and cell death. Finally, incorporating recent research advances, we discuss the current challenges and future prospects in this field, with particular emphasis on intervention strategies targeting Ca²⁺ transport at MAMs, mitochondrial quality control, endoplasmic reticulum stress, and metabolic coupling, as well as their potential for clinical translation. Overall, a deeper understanding of the functional interaction network among mitochondria, the endoplasmic reticulum, and their associated MAMs is expected to provide a robust theoretical foundation and translational insights for elucidating novel molecular mechanisms underlying lung injury and optimizing therapeutic strategies.

**Figure:**
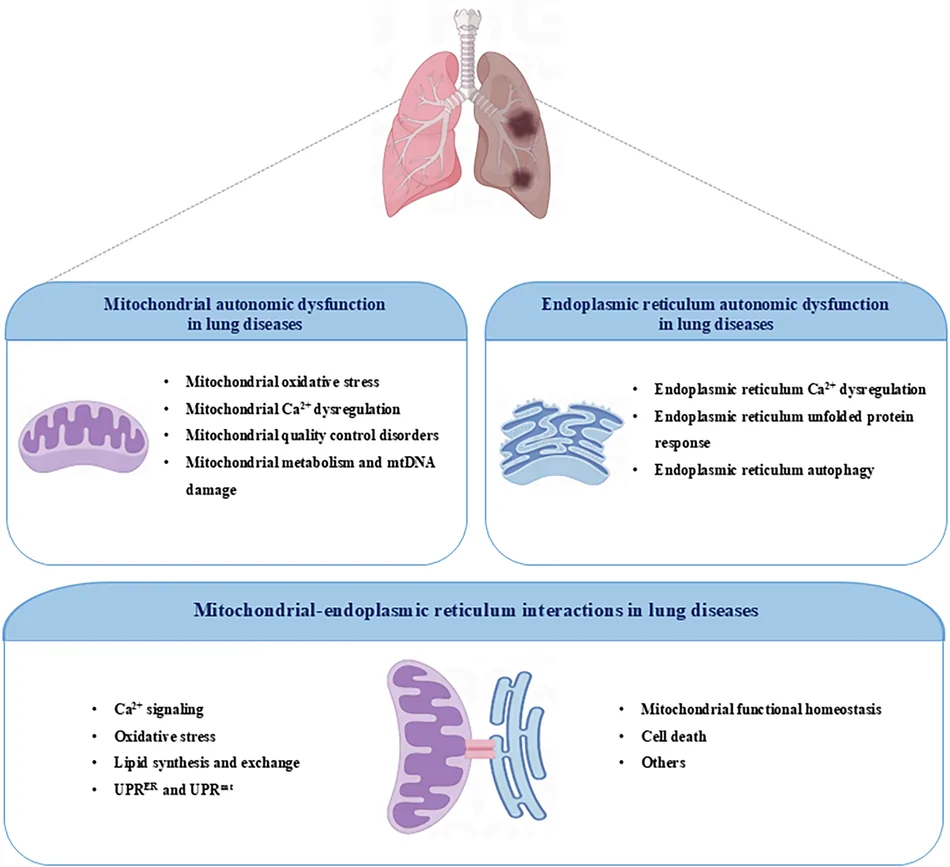

## Facts and questions

1. The absence of lung tissue-specific molecular markers and in situ molecular capture methods for mitochondria-associated membranes (MAMs), combined with the considerable heterogeneity in MAMs abundance and function across distinct lung cell populations, underscores the need for further in-depth investigation.
2. The functions of MAMs exhibit a “double-edged sword” nature: excessive Ca²⁺ transfer from the ER to mitochondria can lead to mitochondrial Ca²⁺ overload and a burst of ROS, while inhibiting Ca²⁺ flux hinders ATP production and tissue function recovery. Therefore, simply enhancing or suppressing MAMs function is unlikely to achieve optimal therapeutic outcomes.
3. Research on MAMs faces significant technical bottlenecks, including limited resolution in vivo imaging and a lack of highly specific probes. Future efforts should focus on developing reversible, non-invasive molecular probes with high temporal resolution, and integrating optogenetic and chemogenetic strategies to achieve precise regulation and construct a “spatial-temporal map” of MAMs in the context of lung injury.

## Introduction

Lung injury-related diseases represent a persistent and severe challenge to global public health and are among the leading causes of acute and chronic respiratory failure, as well as high mortality [1]. According to epidemiological data, acute respiratory distress syndrome (ARDS), acute lung injury (ALI), pulmonary fibrosis, and virus-induced pneumonia account for millions of cases worldwide annually, with mortality rates remaining as high as 30–40%. These conditions are particularly prevalent in clinical settings involving severe infections, trauma, shock, and toxic exposure [2]. Furthermore, they are characterized by diffuse alveolar damage, disruption of the air-blood barrier, and persistent inflammatory responses, all of which contribute to the progressive deterioration of lung function and substantially elevate the risk of mortality [2–6]. In recent years, accumulating evidence suggests that the onset and progression of lung injury are driven not only by inflammatory imbalance and barrier disruption, but also by impaired organelle homeostasis, including key pathological processes such as calcium dysregulation, oxidative stress, and energy metabolism disorders [7, 8]. Notably, mitochondria, the endoplasmic reticulum (ER), and their interactions play a crucial role in orchestrating these stress responses; however, their specific contributions across different types of lung injury remain to be systematically elucidated.

Mitochondria and the ER serve as core executors of lung function homeostasis. Under physiological conditions, mitochondria, as the central hub of energy metabolism, supply ATP to cells with high metabolic demands, such as alveolar epithelial cells and pulmonary microvascular endothelial cells, and contribute to the maintenance of redox homeostasis and the regulation of Ca^2+^ signaling. Concurrently, mitochondria preserve the structural integrity and functional stability of the mitochondrial network through mitochondrial quality control (MQC) mechanisms, encompassing the dynamic balance between mitochondrial fission and fusion, as well as mitophagy [9]. The ER also plays an indispensable role in maintaining cell homeostasis. Structurally, the ER comprises an interconnected network of sheet-like and tubular membrane structures distributed throughout the cytoplasm. It serves critical functions in Ca^2+^ storage and release, protein folding and processing, and ER-phagy, thereby maintaining intracellular protein homeostasis [10, 11]. However, under pathological conditions such as ALI, infection, and fibrosis, mitochondria exhibit ultrastructural damage, impaired respiratory chain function, increased membrane permeability, increased reactive oxygen species (ROS) production, and impaired mitochondrial quality control (MQC), including dysregulated mitophagy and fission–fusion dynamics. Together, these alterations promote apoptosis and inflammatory responses, thereby driving the progression of lung injury [12, 13]. In the ER, disruption of Ca²⁺ homeostasis, increased protein folding load, and impaired ER-phagy thereby activate the unfolded protein response (UPR) and induce the upregulation of molecular chaperones such as immunoglobulin-binding protein (BiP)/glucose regulated protein 78 (GRP78) to mitigate the accumulation of misfolded proteins and exert cytoprotective effects [14]. However, when ER stress is prolonged or excessive, it leads to proteostasis imbalance, enhanced apoptosis, and ultimately drives disease progression.

Membrane contact sites (MCSs) are specialized, closely apposed regions formed between the membranes of two organelles. They maintain their structural integrity through resident protein complexes and lipid microenvironments, and mediate the transport of substances and signals [15, 16]. As an important structural platform for inter-organelle communication, the ER forms both binary and tripartite membrane contact sites with multiple organelles, including lipid droplets [17], which regulate lipid storage; lysosomes and peroxisomes [18, 19], which coordinate metabolic flux and autophagy; the Golgi apparatus [20], which contributes to lipid composition remodeling; and the plasma membrane [21], which mediates Ca²⁺ signaling. Moreover, tripartite junctions such as ER-mitochondria-lipid droplet [22] and ER-mitochondria-lysosome [23] interfaces have also been identified, serving as complex hubs for signal integration. Among these, the ER-mitochondrial contact site is the most extensively studied type of MCS. ER-mitochondrial contact sites are commonly referred to as mitochondria-associated membranes (MAMs) or mitochondria-ER contacts (MERCs) [24]. Ultrastructural studies have demonstrated that the ER and mitochondria are closely apposed through electron-dense tethering structures. The intermembrane distance is approximately 10-30 nm for smooth ER and 20-25 nm for rough ER [25, 26]. Recent molecular dynamics studies have revealed that MAMs are highly dynamic ER-mitochondria contact sites that undergo continuous remodeling, with molecular binding durations ranging from tens of seconds to several minutes. This dynamic characteristic provides a common mechanistic basis for the diverse pathological features of lung injury-related diseases [27]. Functionally, MAMs are considered highly organized platforms that participate extensively in several key cellular processes, including Ca^2+^ signaling, redox homeostasis, lipid synthesis and exchange, coordination of the endoplasmic reticulum unfolded protein response (UPR^ER^) and mitochondrial unfolded protein response (UPR^mt^), and regulation of mitochondrial biogenesis. However, under pathological conditions, MAMs homeostasis is disrupted, manifesting as imbalances in Ca^2+^ signaling, excessive ROS production, UPR dysfunction, and activation of cell death programs. Nevertheless, the regulatory mechanisms governing these structures through specific tethering proteins and signaling pathways remain incompletely understood. Moreover, how these regulatory processes give rise to distinct or even opposing phenotypes in different pathological contexts remains a key question requiring systematic investigation.

As a gas exchange organ directly exposed to the external environment, the lung is dependent on the integrity of the air-blood barrier and the coordinated regulation of macrophages, fibroblasts, and stromal cells. The structure and function of the air–blood barrier depend on energy supply from mitochondrial oxidative phosphorylation (OXPHOS) and the protein folding of the ER [28, 29]. During inflammation and tissue repair, macrophages and fibroblasts are also influenced by mitochondrial metabolic reprogramming [30]. Meanwhile, the lungs are chronically exposed to inhaled pathogens, toxic particles, and oxidizing gases, leading to a higher susceptibility to endoplasmic reticulum stress (ERS) and mitochondrial oxidative damage compared with other organs. As structural hubs coordinating Ca²⁺ signaling, redox balance, and lipid metabolism, the MAMs represent a common pathological basis for various lung injury-related diseases, including ARDS, ALI, pulmonary fibrosis, and viral pneumonia [31, 32]. Based on this, this review focuses on the fundamental mechanisms by which mitochondria and the ER are involved in lung injury, and then examines the evidence for mitochondria-ER communication in these diseases. In addition, drawing on the latest research advances, we explore potential therapeutic strategies targeting mitochondria-ER interactions and their future research prospects, thereby providing new insights and directions for the treatment of lung injury-related diseases.

A systematic literature search was conducted across PubMed, Web of Science, and CNKI databases, encompassing all articles published from inception to February 2026. Search terms included “lung injury”, “mitochondria”, “endoplasmic reticulum”, “mitochondrial-associated membrane”, “mitochondrial-endoplasmic reticulum contact sites”, and “targeted therapy”. The inclusion criteria were as follows: (1) studies investigating the regulation of mitochondrial or ER homeostasis and their interaction mechanisms in lung injury; (2) original research articles published in peer-reviewed journals; and (3) studies involving preclinical and clinical targeted therapies. Studies were excluded if they were duplicated, had insufficient evidence, or were not directly related to lung pathology.

## Mitochondrial autonomic dysfunction in lung diseases

**Fig. 1:**
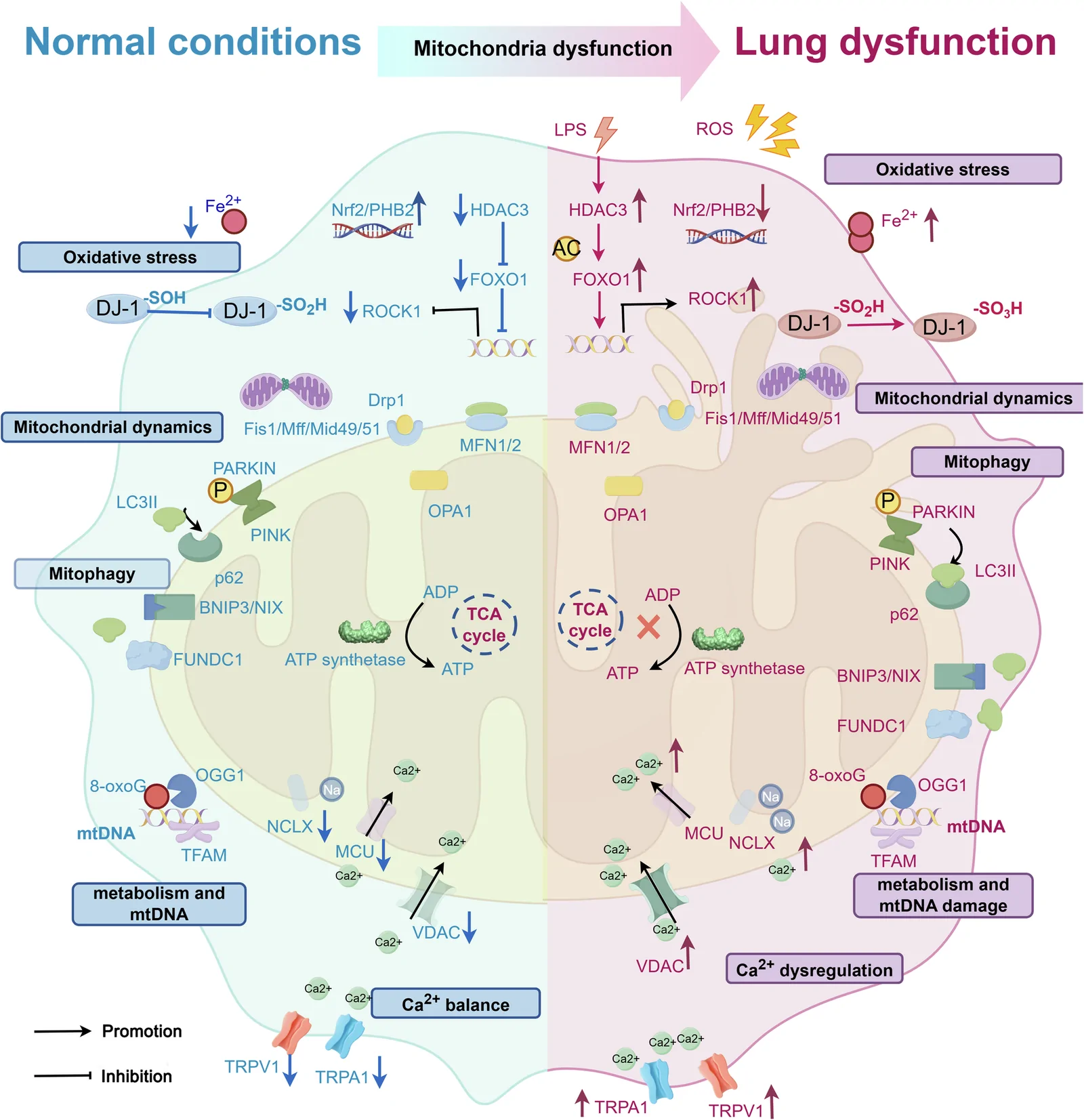
Mitochondrial autonomic dysfunction in lung diseases. During lung injury, multiple factors lead to mitochondrial dysfunction and ROS generation. Oxidative stress induces significant mitochondrial damage and disrupts the homeostasis of the mitochondrial network controlled by MQC, further leading to mitochondrial metabolism and mtDNA damage. Among them, MQC plays a core role in maintaining mitochondrial homeostasis and ensuring normal lung function by regulating adaptive responses such as mitochondrial fusion, fission, and mitochondrial autophagy.

Mitochondria, as core organelles responsible for cellular energy metabolism, redox regulation, and stress sensing, possess highly dynamic membrane structures and metabolic flexibility [33]. Emerging evidence indicates that mitochondrial dysfunction exhibits disease-specific pathological features. For example, in chronic obstructive pulmonary disease (COPD), cigarette exposure induces excessive oxidative stress in alveolar epithelial cells and disrupts mitophagy homeostasis [34]. In idiopathic pulmonary fibrosis (IPF), impaired mitochondrial biogenesis and metabolic reprogramming collectively drive the abnormal transformation of fibroblasts into myofibroblasts and excessive collagen deposition [35]. ARDS and ALI are characterized by mitochondrial bioenergy deficiency, inflammatory infiltration, and disruption of the blood-air barrier integrity [12, 36]. However, these conditions share common features, including Ca²⁺ homeostasis imbalance, excessive ROS generation, and dysregulated MQC. Based on this, this section focuses on the mitochondrial autonomous dysfunction in lung injury, defined as mitochondrial-intrinsic damage events independent of the ER, including oxidative stress, Ca^2+^ imbalance, MQC disorders, and mitochondrial metabolism and mitochondrial DNA (mtDNA) damage, thereby highlighting its significant potential as a therapeutic target. (Fig. 1; Table 1).

**Table 1:** Mitochondrial autonomic dysfunction in lung diseases.

Feature	Mechanisms	Models	Ref.
**Mitochondrial oxidative stress**	Nrf2/PHB2, MMP↓	SA-ALI patients and mice; A549 cells	[41]
	ROS; DJ-1 Cys106-SO₃⁻↑	Human ATII cells	[42]
	SIRT3, SLC7A11, ROS, MDA, GPX4 ↑ ; GSH, Mn-SOD↓	VILI mice; A549 cells	[44]
	xCT and GPx4↓	CSE-exposured mice; BEAS-2B cells	[50]
			[46]
	FBXL5, MMP ↓ ; MitoSOX, IREB2/MFRN2↑	Pulmonary fibrosis mice; MLE-12 cells, and primary AECII	[49]
	SIRT3↓	COPD mice	[43]
	SIRT3, Nrf2, SOD2 ↓ ;ROS↑	Primary hepatocytes from CCl4-injured mice; t-BHP-treated AML12 cells	[51]
	ROS ↑ ; SENP1/SIRT3, SOD2↓	AT2 pulmonary fibrosis	[45]
**Mitochondrial Ca**^**2+**^ **dysregulation**	Ca^2+^↑	LPS-induced ALI mice; MH-S cells	[61]
	Ca^2+^↑	Pulmonary hypertension rats; RV myocytes	[58]
	Ca^2+^, TRPA1, TRPV1↑	CSE-induced A549 cells; BEAS-2B cells	[57]
**Mitochondrial quality control disorders**	p-Drp1 S635; p-Drp1 S656↑	LPS-induced BMM; BMDMs cells	[67]
	p-Drp1/Drp1 ↑ ; OPA1↓	PM2.5-induced lung injury in mice; A549 cells	[66]
	CERK/ARNTL, MFN2 ↓ ; Drp1, MFF↑	FLYDWC50-1C hypobaric hypoxic cabin established HAPM mice model; AT1 cells	[68]
	SIRT1 SUMO1, OPA1, MFN1, MFN2↓	SENP1^flox/-^mice; Sftpc-Cre^+/+^; Hyperoxia-induced HAECs cells	[69]
	Trx2/Myo19/p-Drp1, Fis1↑	LPS-induced ARDS mice; Human AT II cells;	[71]
	S-OPA1 ↑ ; L-OPA1↓	smokers, COPD and IPF lung tissues; CS-induced mice; Primary lung fibroblasts; naked mole rat (NMR) lung fibroblasts, human fetal lung fibroblast (HFL1), mouse embryonic fibroblast; BEAS-2B cells	[72]
	Drp1 ↑ ;Nrf2↓	LPS-induced ALI mice; AEC II cells	[70]
	HDAC3/FOXO1/ROCK1, Drp1, Fis1, LC3II/LC3I, Parkin, PINK1, Pgc-1 α, Cpt1a, Mcad, Acsm2, and Acat1 ↑ ; OPA1, p62 ↓ ;	LPS-induced ALI mice; primary AT2 cells	[13]
	Beclin, LC3-II, SQSTM1/p62↓	Staphylococcus aureus sepsis mice	[80]
	LC3-II ↑ ; p62↓	Cl_2_-induced lung injury in mice; NCl-H441 cells	[81]
	PINK1/PARK2↓	COPD patients; CSE-induced BEAS-2B cells	[82]
	PRKN ↓ ; PINK1, RIP3↑	COPD patients; CSE-induced lung injury in mice ; BEAS-2B cells	[84, 85]
	TFEB, LAMP1, Beclin, and LC3B ↓ ; p62/SQSTM1↑	LPS-induced ALI rats; primary alveolar type II epithelial cells	[78, 79]
	SIRT1, LC3 II, LC3, Beclin1, ATG5, and ATG7↓	CSE-induced MLE-12 cells	[83]
	P62, LC3B-II↑	PS-NP-induced BEAS-2B cells	[46]
**Mitochondrial metabolism and mtDNA damage**	OCR, ATP, GLUT1, glutamine ↓ ; γ-H2AX, lactate↑	BLM-induced MLE12 cells	[94]
	AMPK ↑ ; ADP/ATP, PD-L1, TGF-β ↓	Radiation induced-pulmonary fibrosis mice	[95]
	ΔΨm, p-Drp1 (Ser616), MFF, mtDNA, ND2, COX2, OXPHOS complexes I, II, III, IV and V enzymes, EGFR ↑ ; MFN1/2↓	SARS-CoV-2-induced micel; A549 and Galu cells	[96]
	mtDNA ↑ ; Ogg1↓	Rat PAECs	[98]
	mtDNA-D-loop, TFAM↑	Rat PAECs	[97]
	mtDNA ↑ ; OGG1, BRR, MRR, ATP↓	Cl2-induced lung injury in mice; NCI-H441 cells	[99]
	mtDNA, NLRP3, cGAS-STING↑	CLP-induced sepsis mice	[100]

### Mitochondrial oxidative stress

Oxidative stress is one of the earliest mitochondrial pathological events in lung injury, characterized by redox imbalance and excessive ROS production, which cause oxidative damage to cellular proteins, lipids, and nucleic acids, ultimately leading to signaling dysfunction and cell death [37]. As the main source of endogenous ROS, mitochondria generate excessive ROS in response to stimuli such as mechanical stress, pollutant exposure (e.g., PM2.5), infection, and inflammation, thereby further aggravating oxidative damage [38, 39]. Lung epithelial cells are highly sensitive to ROS, and levels of DNA oxidation products (e.g., 8-hydroxydeoxyguanosine) are significantly elevated in both COPD and IPF [40]. Moreover, ROS increases pulmonary microvascular endothelial permeability, thereby exacerbating pulmonary edema and inflammatory responses.

Multiple intracellular protective pathways are involved in defense against ROS and play crucial roles in lung injury. Nuclear factor erythroid 2–related factor 2 (Nrf2) is negatively correlated with disease severity in sepsis-associated ALI, whereas pharmacological activation of the Nrf2- prohibitin2 (PHB2) axis helps maintain mitochondrial structure and function [41]. Oxidative stress can also induce conformational changes in key protective proteins. For example, the antioxidant protein DJ-1 is oxidized from Cys106-SO_2_^−^ to Cys106-SO₃⁻ under conditions of excessive oxidative stress, and its inactivation has been confirmed in smokers and emphysema models [42]. The mitochondrial deacetylase Sirtuin 3 (SIRT3) maintains redox homeostasis by promoting SOD2 deacetylation and reducing FOXO3 acetylation levels. Consistently, SIRT3 overexpression effectively scavenges mtROS and delays alveolar epithelial type II cells (AECII) senescence [43, 44], whereas SIRT3 deficiency exacerbates BLM or CdCl₂-induced oxidative damage [45]. The upstream regulatory factor SENP1 sustains SIRT3 activity, and its inhibition further aggravates mtROS accumulation and alveolar epithelial injury.

Notably, oxidative stress and iron homeostasis dysregulation frequently interact and jointly contribute to the progression of lung injury. Elevated levels of cytoplasmic free iron promote ROS generation *via* the Fenton reaction, leading to mitochondrial damage and triggering ferroptosis [46–48]. In a BLM-induced pulmonary fibrosis model, EP4 agonist was shown to reduce mitochondrial iron deposition in AECII *via* the FBXL5-IREB2-MFRN2 axis [49]. During hemolytic lung injury, heme-induced ROS production and ferroptosis also represent key pathological mechanisms. In addition, pharmacological activation of SIRT3 exerts anti-ferroptosis effects by inhibiting lipoxygenase activity, and is considered a potential therapeutic target [50–52].

### Mitochondrial Ca^2+^ dysregulation

Mitochondria serve as the central hub of intracellular Ca²⁺ homeostasis, and their dysregulation constitutes a critical mechanism driving lung injury. Under physiological conditions, Ca^2+^ enters the intermembrane space through voltage-dependent anion channels (VDACs) in the outer membrane (OMM), and is subsequently taken up by the mitochondrial calcium uniporter (MCU) located in the inner membrane (IMM), thereby facilitating OXPHOS and metabolic regulation [53]. Conversely, mitochondrial Ca²⁺ efflux is primarily mediated by the mitochondrial Na^+^/Ca^2+^ exchanger (NCLX) and the mitochondrial permeability transition pore (mPTP) [54]. Meanwhile, transient receptor potential (TRP) channels, such as TRPA1 and TRPV1, which are widely expressed in the respiratory tract, directly mediate extracellular Ca^2+^ influx [55, 56]. However, under pathological conditions, Ca²⁺ overload leads to a decrease in mitochondrial membrane potential, impaired OXPHOS, and programmed cell death, thereby serving as a key factor driving lung injury.

Experimental evidence demonstrates that cigarette smoke extract (CSE) induces intracellular and mitochondrial oxidative stress and activates TRPA1/TRPV1-mediated Ca²⁺ influx, while its inhibition or gene knockout reduces oxidative stress and blocks Ca²⁺ influx [57]. In both pulmonary arterial hypertension (PAH) and ALI, elevated cytoplasmic and mitochondrial Ca²⁺ level are closely associated and contribute to endothelial dysfunction, immune activation, and pulmonary vascular remodeling [58–60]. Mitochondrial damage in macrophages further disrupts Ca²⁺ signaling, thereby exacerbating lung injury [61]. In summary, mitochondrial Ca²⁺ abnormalities and TRP channel-mediated Ca²⁺ influx collectively constitute key pathological mechanisms underlying pulmonary Ca²⁺ imbalance.

### Mitochondrial quality control disorders

MQC maintains mitochondrial homeostasis by coordinating mitochondrial fusion and fission, biogenesis, and mitophagy, and represents a key mechanism for ensuring normal pulmonary function [62]. MFN1/2 and optic atrophy 1 (OPA1) regulate outer and inner mitochondrial membrane fusion [63, 64], while Drp1, together with mitochondrial fission 1 protein (Fis1), mitochondrial fission factor (MFF), and mitochondrial dynamics protein 49/51 (MiD49/51), drives mitochondrial fission [65], thereby maintaining a dynamic balance in mitochondrial morphology and function.

Studies have shown that various lung injury-related stimuli, such as LPS, silver nanoparticles, PM2.5, and CSE, trigger mitochondrial dynamic imbalance by upregulating Drp1 (mouse Ser635/human Ser616), which is manifested as mitochondrial fragmentation and decreased membrane potential [66, 67]. Mechanistically, downregulation of the circadian rhythm factor ARNTL [68] and suppression of SIRT1 stability [69] have been confirmed to contribute to this process. Conversely, inhibition of mitochondrial fission [70] can alleviate mitochondrial dysfunction [57]. Furthermore, pharmacological activation of the Trx2-Myo19/p-Drp1 pathway-dependent mitochondrial fission promotes the clearance of damaged mitochondria and protects against ALI induced by ARDS [71]. These findings suggest that mitochondrial fission plays a context-dependent role in lung injury. Excessive or sustained Drp1 activation appears to be detrimental, whereas moderate fission may facilitate the clearance of damaged mitochondria and thus confer protective effects. Histone deacetylase 3 (HDAC3) regulates MQC through ROCK1 and its transcription factor FOXO1, and its inhibition alleviates LPS-induced sepsis-associated ALI [13]. In patients with COPD, the conversion of long OPA1 to short OPA1 is closely associated with mitophagy and mitochondrial dysfunction, and is considered a potential novel therapeutic target [72].

Mitophagy, as an important component of MQC, primarily mediates the clearance of damaged mitochondria through PTEN-induced putative kinase 1 (PINK1)-parkin RBR E3 ubiquitin protein ligase (Parkin)-dependent pathways, as well as PINK1/Parkin-independent pathways such as BNIP3/NIX and FUN14 domain-containing protein 1 (FUNDC1) [73, 74]. In most cases, autophagy induction serves as a cytoprotective mechanism; however, insufficient or excessive mitophagy may exacerbate lung injury [75–77].

Moderate activation of mitophagy facilitates the clearance of damaged mitochondria and maintains cellular homeostasis. For example, TFEB overexpression enhances mitophagy by upregulating p62/SQSTM1, thereby alleviating LPS-induced ALI [78, 79]. Similarly, under *Staphylococcus aureus*-induced sepsis or chlorine exposure conditions, the autophagy activator trehalose significantly improves lung injury, while the autophagy inhibitor 3-methyladenine exacerbates tissue damage [80, 81]. However, excessive mitophagy caused by severe or prolonged stress may further aggravate tissue injury [46]. Notably, PINK1-Parkin-mediated mitophagy exhibits a bidirectional regulatory role in CS-related lung injury. On the one hand, PINK1-Parkin signaling accelerates fibrosis progression in CS-induced COPD [82–84]. On the other hand, high concentrations of CS induce PINK1 accumulation, leads to excessive activation of mitophagy and consequent exacerbation of cellular damage. This mechanism has been supported by studies involving PINK1 loss of function [85]. These divergent effects may be associated with differences in the threshold of mitochondrial damage. In addition, PINK1 not only induces mitophagy in response to CS exposure, but also exacerbates the loss of mitochondrial membrane potential (MMP). The mitochondrial division inhibitor Mdivi-1 can suppress excessive mitophagy, suggesting that mitophagy and mitochondrial structural integrity jointly determine the ultimate fate of CS-exposed cells. Furthermore, this bidirectional regulation is closely related to cell types, including immortalized cell lines and primary AECII, as well as the stage of disease progression.

### Mitochondrial metabolism and mtDNA damage

Mitochondria generate ATP through the tricarboxylic acid (TCA) cycle, fatty acid β-oxidation, and OXPHOS, thereby serving as the central hub of cellular energy metabolism [86]. This process inevitably produces mtROS [87]. At physiological levels, mtROS enhance antioxidant defenses through the “mitohormesis effect”; however, excessive accumulation damages mtDNA, lipids, and proteins, leading to electron transport chain uncoupling and mitochondrial dysfunction. Human mtDNA is a circular genome 16,569 bp that encodes 13 OXPHOS proteins, 22 tRNAs, 2 rRNAs [88]. Its integrity is indispensable for maintaining mitochondrial function. The base excision repair (BER) pathway represents the primary mechanism counteracting mtDNA oxidative damage [89–92], within which hOGG1 plays a critical role in the removal of 8-oxoguanine lesions. Furthermore, mitochondrial transcription factor A (TFAM) protects mtDNA by binding to it; however, aberrant binding leads to mtDNA exposure and degradation, subsequently triggering damage signaling [93].

Various lung injury models have revealed the crucial role of mitochondrial metabolic dysfunction and mtDNA damage in disease progression. For example, BLM exposure inhibits mitochondrial respiration in lung epithelial cells, reduces glucose metabolism, and induces compensatory enhancement of glutamine metabolism, thereby maintaining the TCA cycle and mitigating DNA damage and cell death [94]. Targeted regulation of OXPHOS or inhibition of glycolysis can reverse hypoxia-associated metabolic disturbances, thereby attenuating pulmonary fibrosis [95]. However, in viral infections, SARS-CoV-2 induces abnormal ATP accumulation and metabolic dysfunction by enhancing OXPHOS and EGFR mitochondrial translocation [96]. This difference mainly arises from variations in the type of injury, pathogen characteristics, and host response patterns, reflecting the metabolic plasticity of mitochondria as the cellular energy hub under different pathological conditions. Meanwhile, abnormal mtDNA damage and repair are closely linked to inflammatory responses in various lung injury models. hOGG1 overexpression reduces apoptosis of pulmonary artery endothelial cells under oxidative stress or hypoxia [97, 98], while its deficiency exacerbates inflammatory response [99]. Intraperitoneal injection of mtDNA promotes its release into the cytoplasm *via* mPTP and VDAC, activating the NLRP3 and cGAS-STING pathways, and thereby promoting pathological changes in ALI/ARDS [100].

## Endoplasmic reticulum autonomic dysfunction in lung diseases

**Fig. 2:**
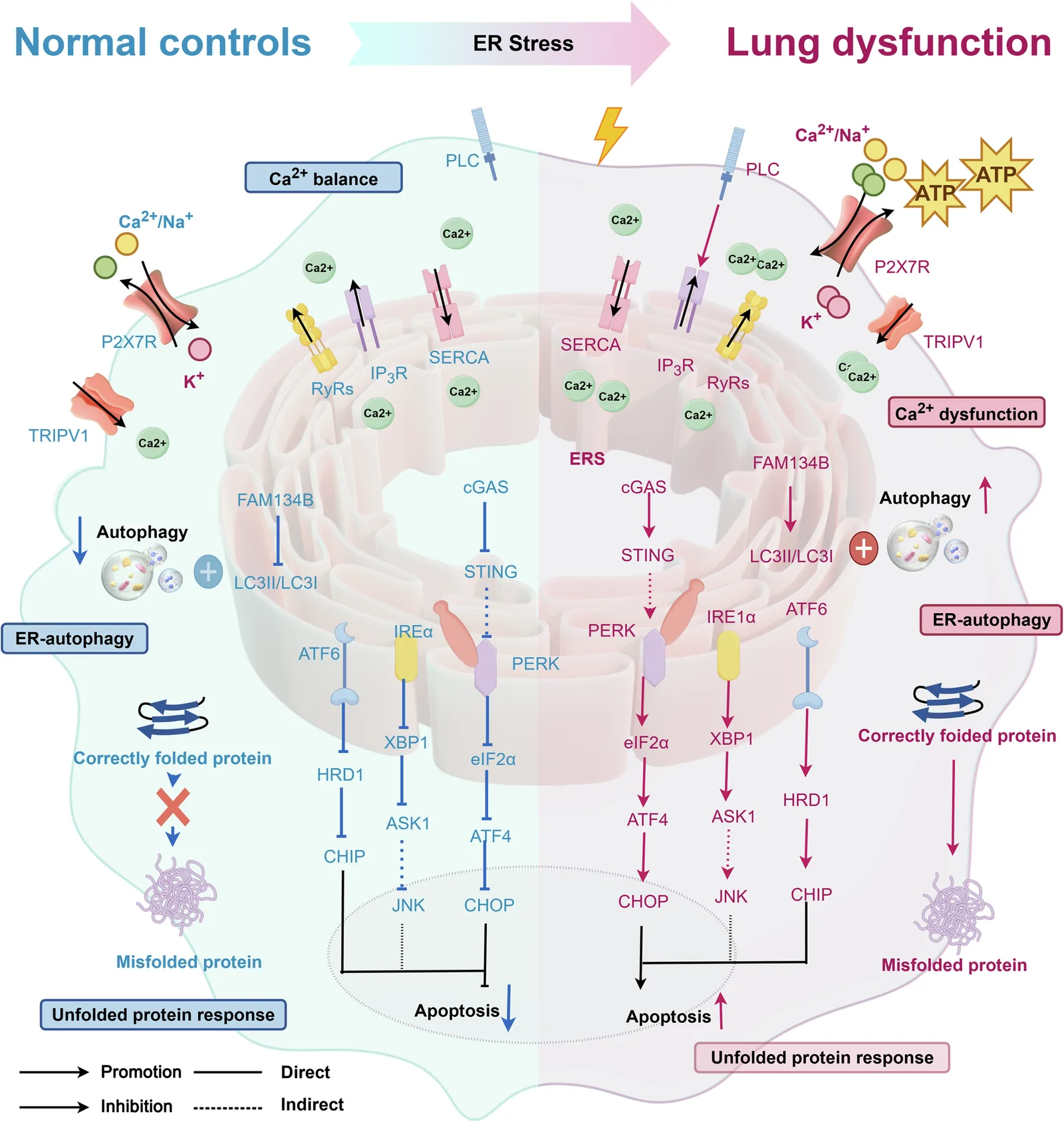
Endoplasmic reticulum autonomic dysfunction in lung diseases. During lung injury, ER dysfunction induces cellular Ca^2+^ imbalance and ERS. During lung injury, overactivation of IP_3_R and RyRs responsible for ER Ca^2+^ efflux and impaired SERCA function of ER Ca^2+^ influx leads to an imbalance of ER Ca^2+^ homeostasis. Simultaneously, the accumulation of unfolded or misfolded proteins in the ER lumen induces ERS and activates the UPR. During lung injury, all three UPR signaling pathways are active, including PERK, IRE-1α, and ATF6. The PERK-eIF2α-ATF4/CHOP pathway inhibits translation and promotes apoptosis. Unspliced XBP1 is converted into spliced XBP1 (XBP1s), which then regulates folding and degradation genes. ATF6 cleavage and activation regulate molecular chaperones and ERAD genes. The PERK-eIF2α-ATF4/CHOP pathway inhibits translation and promotes apoptosis. Unspliced XBP1 is converted into spliced XBP1 (XBP1s), which then regulates folding and degradation genes. ATF6 cleavage and activation regulate molecular chaperones and ERAD genes. Transient activation helps relieve stress, while sustained or intense stress promotes the UPR to shift to pro-apoptotic pathways, thereby initiating inflammatory responses and pathways leading to cell death.

The ER is responsible for the synthesis, folding, and structural control of more than one-third of cellular proteins [101], and also serves as a central organelle for Ca^2+^ storage and signal transduction [101, 102]. Under stress conditions, misfolded proteins are cleared through ER-associated degradation (ERAD). However, persistent accumulation of misfolded proteins leads to ERS, which subsequently activates UPR. The UPR restores cellular homeostasis by attenuating protein synthesis, regulating gene expression, and determining cell fate, including apoptosis. ERS caused by protein homeostasis imbalance has been widely reported in various lung injury-related diseases, highlighting its potential as a therapeutic target. This section therefore focuses on the autonomous stress responses of the ER in lung injury, with particular emphasis on three aspects: Ca^2+^ dysregulation, UPR signaling, and ER-phagy (Fig. 2; Table 2).

**Table 2:** Endoplasmic reticulum autonomic dysfunction in lung diseases.

Feature	Mechanisms	Models	Ref.
**Endoplasmic reticulum Ca**^**2+**^ **dysregulation**	PLC/IP_3_R/Ca^2+^↑	Pulmonary hypertension rats; RPASMC cells	[110]
	Ca^2+^, TRPA1, TRPV1↑	CSE-induced A549 cells; BEAS-2B cells	[57]
	P2X7r, TRPV1, Ca^2+^, p-IREAα, p-ASK1↑	ETH, ALD or e-cig-induced hPAEpiC cells	[112]
**Endoplasmic reticulum unfolded protein response**	p-PERK/p-eIF2a, LC3-II, ATG12 ↑ ; ATP↓	CSE-induced HUVECs	[183]
	IRE1α, eIF2α, ATF6α, HRD1, CHIP↑	PS-NPs-induced lung injury mice; BEAS-2B cells	[121]
**Endoplasmic reticulum autophagy**	RETREG1/FAM134B, p38 ↑ ; RIG1↓	SIV-infected piglet model; PAMs	[125]
	P62, LC3B, PERK/eIF2α/ATF4, IRE1, ATF6 ↑ FAM134B↓	LPS-induced sepsis mice; THP-1 cells	[126]

### Endoplasmic reticulum Ca^2+^ dysregulation

Ca^2+^ serves as a crucial second messenger in the ER, where it maintains protein folding, enzyme activity, and signal transduction. Its homeostasis depends on Ca^2+^ uptake mediated by sarcoplasmic/endoplasmic reticulum Ca^2+^-ATPase (SERCA) and Ca^2+^ efflux mediated by inositol 1,4,5-trisphosphate receptors (IP_3_R) and ryanodine receptors (RyR) [103], and is precisely regulated by high-affinity Ca^2+^-binding proteins such as calreticulin and calnexin [104, 105]. Among these, the phosphoinositide-specific phospholipase C (PLC)/IP_3_R pathway represents a classical Ca²⁺ signaling involved in transient Ca^2+^ elevation and signal amplification [106].

Under pathological conditions, P2X7R activation by high concentrations of extracellular ATP promotes Ca^2+^/Na^+^ influx and K^+^ efflux [107], thereby exacerbating both ERS [108] and inflammatory responses [109]. In PASMCs from patients with PAH, excessive activation of the PLC/IP_3_R pathway is associated with sustained elevation of cytoplasmic Ca^2+^ levels, which in turn promotes aberrant cell proliferation [60, 110, 111]. Similarly, TRPV1, a highly selective Ca^2+^ channel, is upregulated in smoking and e-cigarette-related airway inflammation, and its pharmacological inhibition or genetic knockout reduces Ca²⁺ overload and ERS [57, 112]. Current studies have primarily focused on P2X7R- and TRPV1-mediated Ca^2+^ dysregulation, whereas the role of the canonical SERCA-IP₃R-RyR pathway in ER Ca²⁺ homeostasis during lung injury remains insufficiently explored. Systematically elucidating the roles of ER Ca^2+^ regulatory proteins SERCA, IP₃R and RyR in lung injury will contribute to a more comprehensive understanding of ER Ca²⁺ dysregulation and may reveal novel therapeutic targets.

### Endoplasmic reticulum unfolded protein response

The ER is central to protein folding and quality control, and its homeostasis depends on molecular chaperones, ERAD, and the UPR [113, 114]. The UPR is mediated by three principal sensors: protein kinase RNA (PKR)-like endoplasmic reticulum kinase (PERK), inositol-requiring enzyme 1-α (IRE1α), and activating transcription factor 6 (ATF6) [115]. Under physiological conditions, these sensors remain in a quiescent state through association with the chaperone BiP/GRP78 on the ER membrane. Upon the onset of ERS, BiP/GRP78 dissociates from these sensors and preferentially binds to misfolded proteins, thereby activating the UPR to restore protein homeostasis [116]. At the level of canonical UPR signaling, PERK inhibits global protein translation by phosphorylating eukaryotic initiation factor 2α (eIF2α), thereby reducing the influx of nascent proteins into the ER and alleviating the protein-folding burden. In parallel, IRE1α generates the transcriptionally active X-box binding protein 1 (XBP1s) through splicing of XBP1 mRNA, thereby inducing the expression of molecular chaperones and ERAD-related genes to enhance protein folding ability and promote the clearance of misfolded proteins. Under stress conditions, ATF6 dissociates from GRP78 and translocates to the Golgi apparatus, where it is cleaved by site-1 and site-2 proteases (S1P and S2P). The resulting active fragment then enters the nucleus to induce the expression of molecular chaperones and ERAD-related genes, further enhancing ER protein quality control capacity [117–119].

In lung injury, imbalance of the UPR has been widely implicated in inflammation, fibrosis, and cell death. Recent studies have demonstrated that CSE induces excessive accumulation of misfolded proteins in the ER, accompanied by activation of the PERK-eIF2α pathway [119]. siRNA-mediated knockdown of PERK and ATF6 has further demonstrated that polystyrene nanoplastics (PS-NPs) induce ERS and protein ubiquitination by activating the PERK-ATF4-C/EBP-homologous protein (CHOP) and ATF6-HRD1/CHIP pathways, as confirmed in both in vitro and in vivo studies [120, 121]. Notably, HRD1, an E3 ligase involved in ERAD, promotes the degradation of misfolded proteins under ERS conditions [122], while ATF6α contributes to ER homeostasis by facilitating the ubiquitination of unfolded or misfolded proteins [121]. Furthermore, clinical analyses have shown that, PERK activation is associated with endothelial-to-mesenchymal transition in pulmonary microvascular endothelial cells of patients with MDA5^+^ dermatomyositis-associated interstitial lung disease, suggesting that UPR signaling may be contribute to lung tissue remodeling and disease progression [120]. Collectively, these findings indicate that PERK, IRE1, ATF6, and their downstream signaling represent key regulatory nodes in ERS-mediated lung injury and may serve as potential therapeutic targets.

### Endoplasmic reticulum autophagy

ER-phagy is a key process in alleviating ERS and maintaining ER homeostasis, restoring protein quality control by selectively removing damaged ER fragments [123]. Among the identified receptors, FAM134B, also known as RETREG1, is the first mammalian ER-phagy receptor. Its LIR domain recruits LC3-II, while its RHD facilitates fragmentation and degradation of the ER, making it a core mediator of selective ER turnover [124]. Moderate activation of ER-phagy exerts protective effects; however, insufficient or excessive activation may induce type II programmed cell death.

Recent studies have shown that FAM134B-mediated ER-phagy is involved in lung injury and inflammatory responses. Aflatoxin B1 enhances ER-phagy and aggravates lung injury by upregulating FAM134B and p38, while inhibiting RIG-I signaling. Mechanistically, FAM134B-specific inhibitors block the ubiquitination of FAM134B and its interaction with LC3B-II, thereby alleviating lung tissue damage caused by excessive ER degradation [125]. Furthermore, in LPS-treated lung epithelial cells or THP-1 cells, FAM134B gene silencing inhibits ER-phagy and exacerbates ALI [126]. In summary, dysregulation of ER autophagy plays an important role in the development and progression of lung injury.

## Mitochondrial-endoplasmic reticulum interactions in lung diseases

The ER and mitochondria dynamically remodel their structures in response to cellular demands, and this plasticity is essential for maintaining their spatial organization and functional coordination. Notably, the number, distance, and stability of MAMs vary depending on cell type and pathological conditions. In lung injury, MAMs not only regulate mitochondrial-ER interactions through Ca^2+^ signaling and ROS, but also influence cell fate and tissue pathological *via* tethering protein-mediated structural coupling. This section systematically summarizes the key functions of MAMs in lung diseases, including Ca^2+^ signaling, oxidative stress, lipid synthesis and exchange, UPR^ER^ and UPR^mt^, mitochondrial homeostasis, and cell death (Table 3).

**Table 3:** Mitochondrial-endoplasmic reticulum interactions in lung diseases.

Target proteins (location)	Functions	Models	Ref.
IP_3_R(ER/MAM)	Disrupted Ca^2+^ homeostasis, with increased flux from the ER to the cytoplasm and mitochondria.	High tidal volume (HTV)-induced mice;	[259]
IP_3_R(ER/MAM)	Mediated Ca^2+^ transport from ER to mitochondria.	LPS-induced ALI mice; MLE-12 cells	[141]
IP_3_R(ER)-GPR75-VDAC(Mitochondria)	Tether between ER and mitochondria; Ca^2+^ transfer between ER and mitochondria	BLM-accelerated lung aging	
SERCA(MAM/ER)	Regulated Ca^2+^ transport between the cytoplasm and the sarco/ER, mitophagy, inflammation, and mitochondrial function	Influenza A virus infected human lung cells (H1395)	[142]
STIM1 (ER)-Orai1	ER calcium depletion leads to the introduction of extracellular Ca²⁺ into the cell.	LPS-induced ALI mice; PMVECs	[143]
VAPB(ER)-PTPIP51(mitochondria)	Coordinated ER-mitochondrial Ca^2+^ transport and regulated autophagy	*Pseudomonas aeruginosa* AA43 strain-induced cystic fibrosis in mice; IB3-1 cells (CF cells) are human bronchial epithelial cells	[144]
VAPB(ER)-PTPIP51(mitochondria)	Coordinated ER-mitochondrial Ca^2+^ transport and regulated ERS	TGF-β1-induced mouse lung fibroblast (MLG)	[154]
MFN2(mitochondria)-MFN2(ER)-IP_3_R(ER)	Coordinated intracellular Ca^2+^ homeostasis; regulated autophagy; regulated pulmonary hemodynamics and vascular remodeling	Monocrotaline (MCT)-induced PAH rat model; TNF-α-induced PASMCs cells	[157]
IRE1α(MAM)-XBP1(ER)-cMyc	Drived NK cell responses against viral infections and tumors; regulated oxidative phosphorylation	Virus-infected mice; NK cells	[196, 217]
BiP/GRP78(MAM)-Sig1R(ER)	Maintained cell homeostasis, anti-inflammatory and barrier protection	LPS-induced ALI mice; HPAECs	[195]
IRE1α(MAM)-TRAF2-ASK1-JNK (mitochondria)	Regulated apoptosis; ERS; mitochondrial dysfunction	LPS-induced ALI/ARDS mice	[197]
STING(ER)- Drp1(mitochondria)	Regulated mitochondrial fission, inflammation, and pyroptosis	LPS-induced ALI/ARDS mice; BMDMs and AMs; THP-1 cells	[216]
MFN2(mitochondria)-MFN2(MAM)-PERK(MAM)	Regulated ferroptosis, mitochondrial functional homeostasis	As-induced ALI mice; MLE-12 cells	[31]
BCAP31(ER)-Fis1(mitochondria)	Regulated mitochondrial homeostasis, ERS and apoptosis	LPS-induced ALI mice; ATII cells	[149]
PACS2(MAM)-TRPV1	Coordinated mitochondria-ER interactions	IPF patient tissue; MLE-12 cells	[229]
PINK1(mitochondria)-ATF3-ERS	Regulated mitochondrial homeostasis	BLM-induced pulmonary fibrosis in mice; primary human pulmonary alveolar epithelial cells; A549 cells	[158, 188]

### Ca^2+^ signaling

Ca^2+^ homeostasis depends on ER storage and MAM-mediated mitochondrial uptake [127]. MAM-mediated Ca^2+^ transport is determined by two key factors: the structural organization of MAMs and their protein composition. First, the distance between the ER and mitochondria critically influences Ca^2+^ transfer efficiency, which is optimal within a range of approximately 10 ~ 30 nm and declines when the gap either narrows or widens beyond this range [128]. Second, the molecular architecture of MAM-associated proteins also regulates Ca^2+^ flux. Ca²⁺ is released from the ER *via* RyR and IP_3_R, and is subsequently transferred into the mitochondrial matrix *via* the VDAC1–GRP75–IP_3_R tethering complex and taken up by the MCU, thereby sustaining TCA cycle activity and OXPHOS to support ATP production [129–133]. It is estimated that more than 80% of mitochondrial Ca²⁺ originates from MAMs [127, 134]. Furthermore, ER Ca^2+^ replenishment relies primarily on SERCAs, which pump Ca^2+^ into the ER lumen. An alternatively spliced isoform of SERCA1, S1T, lacks Ca^2+^ pumping activity, resulting in ER Ca^2+^ leakage and consequently enhancing Ca²⁺ transfer to mitochondria [135]. Upon depletion of ER Ca^2+^ stores, stromal interaction molecule-1 (STIM-1) senses the decrease in luminal Ca²⁺ and translocates to MAMs regions, where it forms a functional Ca²⁺ channel complex with Orai1 on the plasma membrane, thereby triggering store-operated calcium entry (SOCE) [136, 137]. SOCE not only replenishes ER Ca^2+^ stores, but also facilitates Ca²⁺ transfer to mitochondria *via* MAMs, supporting energy production and cell survival. Collectively, these processes constitute a feedback network that regulates Ca²⁺ homeostasis at MAMs, and the maintenance of this balance is crucial for preserving cellular function.

During lung injury, MAM-mediated Ca^2+^ transport undergoes context-dependent dynamic remodeling, manifesting in two distinct pathological modes: over-coupling and under-coupling. Over-coupling is manifested by an abnormally shortened ER-mitochondrial distance, increased MAMs formation, and excessive Ca²⁺ transport flux. For example, PHMG [138], PS exposure [139], and asthma [140] all lead to an abnormal reduction in the distance between the ER and mitochondria, thereby increasing mitochondrial Ca^2+^ uptake. This structural alteration is typically accompanied by remodeling of MAM-associated proteins, including IP₃R, phosphofurin acid cluster sorting protein-2 (PACS2) and FUNDC1, and is further associated with aberrant expression of regulatory proteins such as STIM1, Orai1, and mitofusin 2 (MFN2), collectively disrupting MAMs stability. Notably, silencing IP_3_R partially reverses these phenotypes [141]. Conversely, structural disruption or reduced connectivity of MAMs results in under-coupling, thereby impairing mitochondrial Ca^2+^ uptake and compromising cellular bioenergetics. Influenza A virus disrupts Ca^2+^ homeostasis by inhibiting SERCA activity, leading to autophagy blockade and mitochondrial dysfunction, while its activator CDN1163 restores autophagic flux and ameliorates mitochondrial dysfunction [142]. Excessive activation of SOCE also contributes to the pathological process. Studies have shown that abnormal expression of STIM1/Orai1 in the ALI model leads to MAMs instability, dysregulated Ca²⁺ signaling, and enhanced cell death. Pharmacological inhibition of STIM1–Orai1 activity by the SOCE inhibitor YM-58483 reduces mitochondrial Ca^2+^ influx, restores MAMs homeostasis, and partially alleviates lung injury [31, 143].

In addition to the canonical IP_3_R-GRP75-VDAC axis-mediated ER-mitochondrial Ca²⁺ transport, the structural stability of MAMs depends on the precise regulation of the vesicle-associated membrane protein B (VAPB)-protein tyrosine phosphatase-interacting protein 51 (PTPIP51) transmembrane tethering complex. VAPB on the ER forms a functional complex with the OMM protein PTPIP51, thereby increasing MAMs contact sites, promoting mitochondrial Ca²⁺ uptake, and regulating the UPR and autophagy by facilitating functional coupling of the IP_3_R-VDAC axis. Conversely, disruption of VAPB weakens cellular stress responses [142, 144]. Notably, unlike conventional MAMs (10 ~ 30 nm), which primarily mediate Ca²⁺ transport, autophagy-related MAMs exhibit an increased inter-organelle distance of approximately 50 nm, accommodating the extension of autophagic membranes [145]. PACS2 is another key regulatory hub located at the MAM interface and plays a central role in maintaining MAM integrity by preserving ER-mitochondrial structural coupling, suppressing ERS, and stabilizing mitochondrial morphology [146]. Loss of PACS2 induces BAP31 cleavage, leading to mitochondrial fragmentation and ER–mitochondrial uncoupling [147]. BAP31, in turn, promotes mitochondrial function by maintaining mtDNA stability and interacting with TOM40 [148] and Fis1 [149, 150], while simultaneously suppressing ERS through its interaction with syntaxin 17 (STX17) [151] and CDIP1 [152] and through activation of SREBP1C [153]. Thus, BAP31 serves as a critical upstream protective factor coordinately regulates mitochondrial homeostasis and ER function.

In cystic fibrosis bronchial epithelial cells infected with *pseudomonas aeruginosa*, the stability of the VAPB-PTPIP51 complex is enhanced, leading to excessively tight ER-mitochondrial coupling, which in turn suppresses autophagy and establishes a positive feedback loop involving autophagy inhibition, UPR^mt^ and inflammasome activation. Overexpression of VAPB-PTPIP51 further confirmed its key role in mediating Ca²⁺ transfer from the ER to mitochondria. Pharmacological inhibition of the MCU partially corrected this process and alleviated Ca²⁺ overload caused by excessive MAMs coupling [144]. These findings directly elucidate the core mechanism by which the VAPB-PTPIP51 complex influences autophagy and inflammation through the regulation of Ca²⁺ homeostasis. Conversely, in fibrotic pathology, reduced VAPB-PTPIP51 levels in activated fibroblasts disrupt MAM integrity, resulting in decreased mitochondrial Ca^2+^ levels and thereby triggering ERS and mitochondrial energy deficiency [154]. These apparently discrepant findings suggest that the VAPB–PTPIP51 axis exerts a context-dependent role in lung injury, in which both excessive and insufficient ER–mitochondria coupling may be pathological by disrupting Ca²⁺ homeostasis, autophagy, and cellular bioenergetics through distinct mechanisms.

MFN is a dynamin-related GTPase. MFN1 is localized to the OMM, while MFN2 is present not only in the OMM but is also enriched at MAMs [155]. Beyond its role in maintaining mitochondrial dynamics homeostasis, MFN2 tethers the ER to mitochondria through heterodimerization with MFN1 or homodimerization with itself, thereby regulating key processes including Ca²⁺ transport as well as lipid and glucose metabolism [156]. Under pathological conditions, MFN2 dysfunction has been shown to be closely associated with impaired Ca²⁺ homeostasis.

**Fig. 3:**
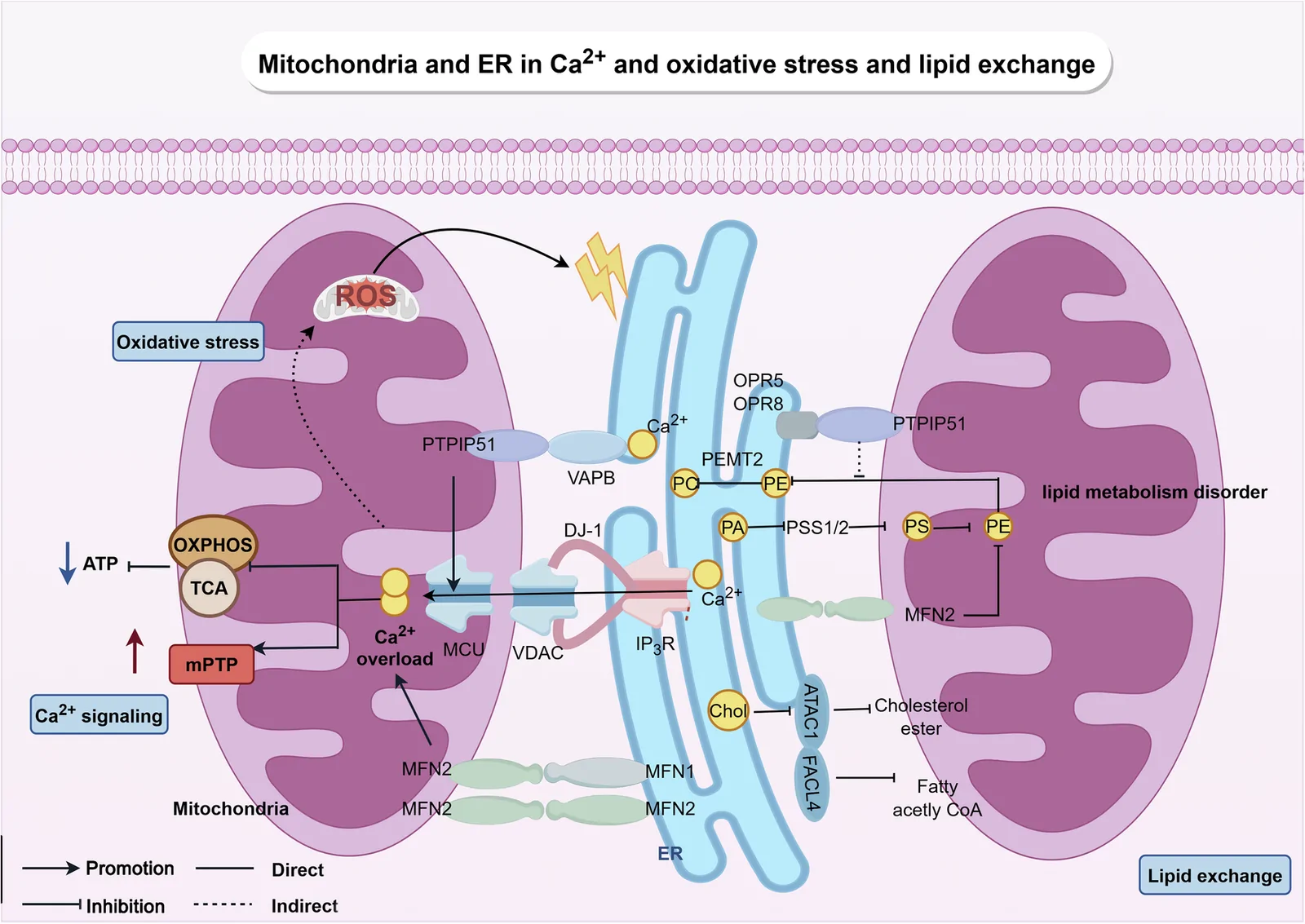
Roles of endoplasmic reticulum-mitochondria communication in Ca^2+^ signaling and oxidative stress and lipid homeostasis. Ca^2+^ signaling. The IP_3_R-GRP75-VDAC complex connects the endoplasmic reticulum and the mitochondrial outer membrane, mediating Ca^2+^ transport to mitochondria, which is further mediated by the MCU of the mitochondrial inner membrane. DJ-1-VDAC, VAPB-PTPIP51, and MFN2 connect the endoplasmic reticulum and mitochondria through homodimerization or heterodimerization with MFN1, and are also involved in the Ca^2+^ transport process. **Oxidative stress:** Mitochondrial dysfunction produces excessive ROS, enhances ERS and promotes Ca²⁺ release, which in turn leads to mitochondrial depolarization and Ca²⁺ overload, further promoting ROS generation. **Lipid synthesis and transport:** After PSS1/2 synthesizes phosphatidic acid (PA) in the ER, phosphatidylserine (PS) is transported to the mitochondria and forms phosphatidylethanolamine (PE) in the endoplasmic reticulum. PE is then transported to the ER where it is converted to phosphatidylcholine (PC) by PEMT2. MFN2, ORP5/8, and PTPIP51 promote trans-MAM lipid exchange. Cholesterol (Chol) metabolism also occurs in MAMs, with ACAT1 catalyzing the esterification of free cholesterol. FACL4 converts long-chain fatty acids into fatty acyl-CoA, making them available for mitochondrial lipid metabolism.

Studies have demonstrated that, in both MCT-induced PAH rat models and in the context of SiO₂ exposure or hypoxia, activation of ERS mediates the downregulation of MFN2 and the upregulation of IP_3_R3, thereby leading to a significant increase in mitochondrial Ca²⁺ levels and a concomitant decrease in cytoplasmic Ca²⁺ levels. These alterations are accompanied by increased expression of ERS markers (ATF6, ATF4, CHOP, and GRP78) and mitochondrial stress-related proteins (PINK1, Parkin, and Drp1), all of which can be reversed by MFN2 overexpression [157, 158]. Notably, the reported changes in ER-mitochondrial contact area induced by MFN2 deficiency remain inconsistent across experimental systems, which may be related to differences in mitochondrial morphology and physiological status [159]. Overall, current evidence suggests that MFN2 maintains Ca²⁺ homeostasis and regulates cell fate through MAMs; however, its precise role in lung injury-related diseases remains to be further elucidated (Fig. 3).

### Oxidative stress

Under physiological conditions, mitochondria and the ER maintain redox homeostasis through MAMs. In pathological processes, excessive ROS production and the resulting oxidative stress represent key drivers of coupled mitochondrial and ER damage. Previous studies have confirmed the existence of a positive feedback loop between Ca^2+^ and ROS [160, 161]. Specifically, mitochondrial dysfunction results in excessive ROS generation, which in turn enhances RyR activity, promotes ER Ca²⁺ release, and sustains activation of UPR. Conversely, ER Ca²⁺ efflux induces mitochondrial depolarization and Ca²⁺ overload, thereby further exacerbating ROS production [162]. Furthermore, excessive ROS generated by respiratory chain complex III amplifies ERS through upregulation of CHOP expression [163]. Taken together, ROS-mediated oxidative stress and Ca²⁺ homeostasis imbalance interact to form the pathological basis of ER-mitochondrial crosstalk, which is highly relevant to lung injury and related diseases.

Extensive experimental studies confirmed the central role of the interaction between ROS and Ca²⁺ in lung injury models. Combined treatment with resveratrol and arsenic trioxide enhances ERS, as evidenced by the upregulation of GRP78 and CHOP, and induces mitochondrial dysfunction in a ROS-dependent manner [164]. In addition, Exposure to SiO₂ nanoparticles triggers excessive ROS production, loss of MMP, and activation of ERS markers [165]. Notably, these effects can be reversed by either MitoQ or the ERS inhibitor salubrinal [31, 166]. Exposure to CSE also induces a biphasic Ca²⁺ response, in which the later peak may be associated with increased ROS levels [167]. Taken together, these findings support a central role of ROS in amplifying ER-mitochondrial crosstalk. Notably, further studies suggest that ERS may precede mitochondrial dysfunction, thereby leading to reduced OXPHOS and increased ROS production. Although this temporal relationship has been demonstrated in the aging heart [168], its relevance to lung injury remains to be further clarified.

### Lipid synthesis and exchange

MAMs serve as a central platform for lipid transport and metabolic regulation. A variety of proteins mediate the non-vesicular transfer of lipids from the ER to mitochondria, including the VAPB-PTPIP51 complex, oxysterol-binding protein-related protein related-protein 5 and 8 (ORP5/8), MFN2, CAV-1, and key biosynthetic enzymes such as phosphatidylserine synthase-1 and 2 (PSS1/2), ACAT1 (also known as SOAT1), and FACL4 (also known as ACSL4). With respect to phospholipid transport, a canonical pathway involves the conversion of phosphatidic acid into phosphatidylserine by PSS1/2 in the ER membrane [169]. Phosphatidylserine is subsequently transported to the IMM, where it is decarboxylated into phosphatidylethanolamine by phosphatidylserine decarboxylase. It is then transferred back to the ER and converted into phosphatidylcholine *via* PEMT2 [170]. This process depends on MAM-localized lipid transport proteins and represents a critical rate-limiting step in maintaining membrane integrity and cellular lipid homeostasis. Mechanistically, ORP5/ORP8 located at MAMs facilitate phosphatidylserine transport through interactions with PTPIP51 [171, 172]. Furthermore, the lipid transfer protein E-Syt1 is recruited into MAMs, where the E-Syt1-PERK complex mediates phospholipid transport between the ER and mitochondria *via* its SMP domain. Disruption of this interaction consequently impairs mitochondrial respiratory function [173, 174]. Moreover, MFN2 directly participates in phospholipid transport, and its dysregulation can alter membrane composition, thereby affecting cellular energy metabolism and contributing to lung injury-related pathologies [175]. In terms of cholesterol and lipid metabolism, ACAT1 is enriched at MAMs and maintains cholesterol homeostasis by catalyzing cholesterol esterification, while FACL4 is involved in fatty acid activation [176]. Loss of FACL4 leads to impaired lipid transport and mitochondrial function, whereas its overexpression suppresses ER-mitochondrial contact formation and mitochondrial respiration and increases sensitivity to ERS [177–180].

In hypoxia-induced PAH mice, mitochondrial phosphatidylethanolamine synthesis, which depends on physical ER-mitochondrial interactions, was significantly impaired, suggesting that MAM-mediated lipid metabolism abnormalities may contribute to pulmonary vascular remodeling and energy metabolism dysfunction [181]. Therefore, fine regulation of MAM-associated proteins is crucial for maintaining lipid homeostasis and inter-organelle communication, although their specific roles in lung pathology require further investigation.

### UPR^ER^ and UPR^mt^

UPR functions through three core pathways: PERK, IRE1α, and ATF6 [115]. Notably, UPR sensors are located at MAMs [182], where they not only mediate canonical UPR signaling to maintain protein homeostasis but also regulate ER–mitochondrial crosstalk through non-canonical pathways, including ER-mitochondrial Ca^2+^ transport, ROS signaling, and energy metabolism [182]. Thereby playing a crucial role in the initiation and progression of lung injury.

In the canonical UPR signaling, PERK inhibits global protein translation by phosphorylating eIF2α while selectively promoting ATF4 expression, thereby reducing the burden of protein folding in the ER [183]. Beyond its canonical role, the PERK/eIF2α/ATF4 axis serves as a critical link between the UPR^ER^ and the UPR^mt^, helping to maintain mitochondrial oxidative phosphorylation (OXPHOS) homeostasis [184]. Moreover, PERK directly regulates Ca²⁺ transport and ROS signal modulation and contributes to the maintenance of MAM integrity [173, 182]. MFN2, as an upstream negative regulator of PERK, restrains excessive PERK activation and suppresses the transfer of excess ROS to mitochondria, thereby maintaining ER-mitochondrial homeostasis. Conversely, MFN2 deficiency results in persistent PERK activation and ultimately causes mitochondrial dysfunction [185]. Collectively, these findings suggest that MAMs are not only a structural hub of ER-mitochondrial communication, but also a critical platform for UPR-mediated non-canonical signaling.

The canonical and non-canonical functions of PERK have been demonstrated in multiple lung injury models. Studies have shown that ATF4-dependent UPR^mt^ enhances the sensitivity of lung epithelial cells to BLM-induced injury, as evidenced by ATF4 knockdown experiments [115]. Notably, UPR^ER^ functions as an upstream signal for UPR^mt^ activation, whereas the UPR^mt^ does not activate UPR^ER^ in return, indicating a directional regulatory relationship between these two pathways [186]. Moreover, ERS inhibitors and ATF4 siRNAs have shown that ERS enhances ATF4 transcriptional activity at the promoter regions of metabolism-related genes, thereby inhibiting OXPHOS and remodeling the cellular metabolic state [187]. ATF3 is also significantly upregulated in IPF patients and BLM-treated mice, where it promotes mitochondrial dysfunction and cellular senescence, and its overexpression further accelerates disease progression [188]. Meanwhile, the scaffold protein Akap1 is involved in ERS-related mitochondrial damage by regulating the PKA/eIF2α/JNK signaling axis, and its deficiency exacerbates hyperoxia-induced ALI [189, 190]. Overall, ER–mitochondrial signal coupling, especially the PERK-ATF4-CHOP axis, constitutes a critical regulatory node in the pathogenesis and progression of lung injury.

IRE1α is the most evolutionarily conserved branch of the UPR. Canonically, it promotes protein-folding capacity and restores ER homeostasis by splicing XBP1 mRNA, thereby inducing expression of the molecular chaperone BiP/GRP78 [191]. In addition to this canonical role, IRE1α located at MAMs is anchored by the ER chaperone Sig-1R, through which it exerts multiple non-canonical functions [192, 193]. This process is modulated by Sig1R stabilization and the mitochondrial ubiquitin ligase MARCH5 [194], which restrains excessive IRE1α activation through ubiquitination and thereby suppresses ERS-induced apoptosis. Moreover, VAPB can also modulate UPR activity through regulation of IRE1α-XBP1 signaling. Taken together, these findings indicate that IRE1α plays a central role in protein homeostasis, energy metabolism, and cell survival.

In LPS-induced endothelial cell injury and ALI models, depletion or inactivation of BiP/GRP78 facilitates the dissociation and activation of Sig1R from BiP/GRP78 at MAMs, thereby promoting endothelial barrier function. Consistent with this, the Sig1R activator PRE-084 significantly attenuates barrier disruption caused by plasma or thrombin in patients with sepsis [195]. In neutrophils infected with *Chlamydia pneumoniae*, TLR2-dependent ERS promotes both upregulation of IRE1α expression and splicing of XBP1 mRNA, and this phenomenon can be reversed by both exhaustion and TLR2 deficiency [196]. Moreover, ERS activates JNK through the IRE1α signaling pathway and induces its translocation to the OMM, where it interacts with the scaffold protein Sab and triggers mitochondrial dysfunction. Selective inhibition of the JNK–Sab interaction using the Tat-SabKIM1 peptide effectively blocks this harmful inter-organelle signaling, thereby mitigating mitochondrial dysfunction and ameliorating lung injury [195, 197]. In addition, H₂O₂ activates the IRE1/eIF2α/ATF4/ATF6 signaling pathways and induces Ca^2+^ overload through ERS [198].

**Fig. 4:**
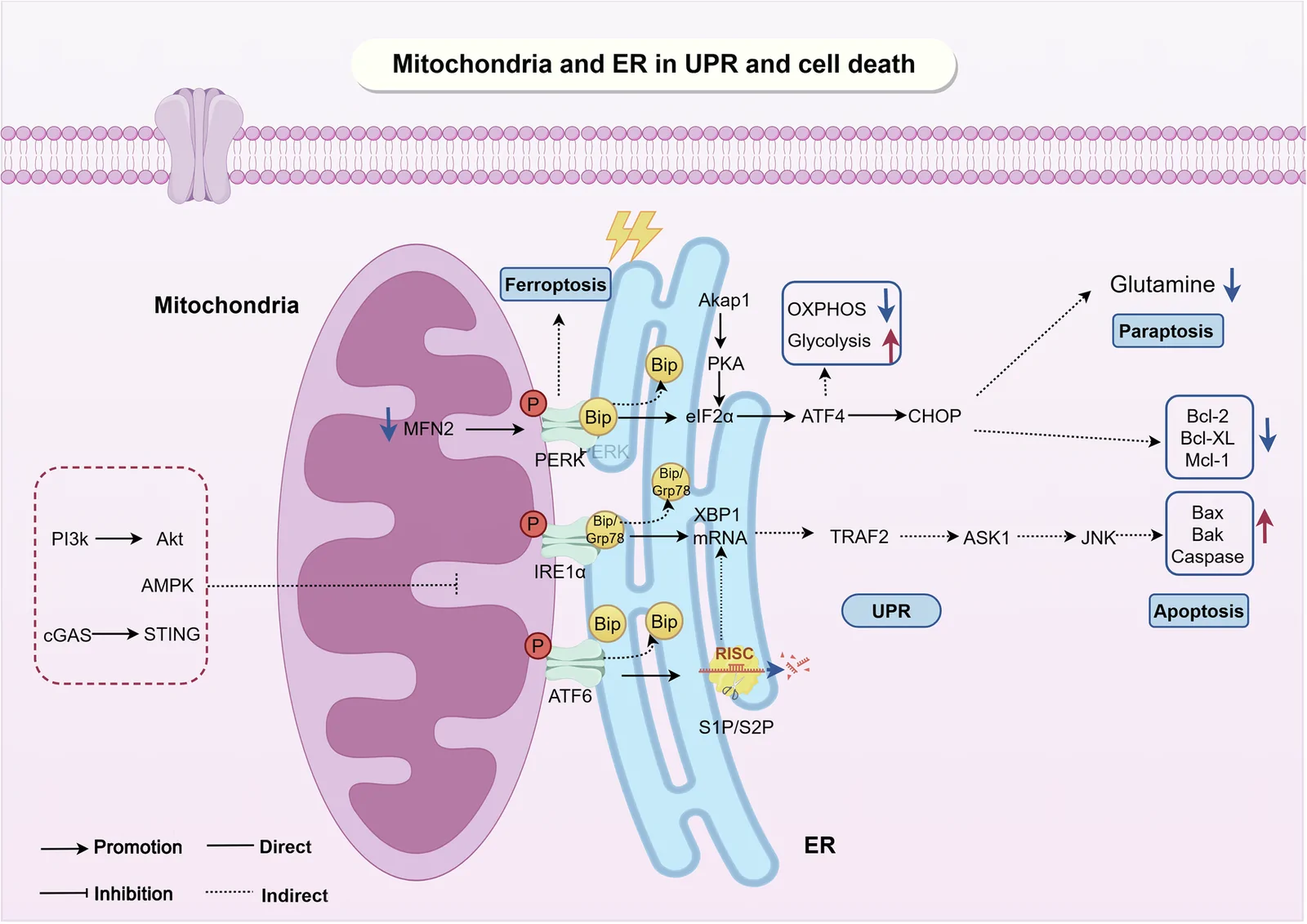
Roles of endoplasmic reticulum-mitochondria communication in UPR and cell death. ERS triggers BiP dissociation from the stress sensors PERK, IRE1α, and ATF6, thereby activating the UPR. PERK phosphorylates eIF2α, thereby initiating the translation of ATF4/CHOP. XBP1 is converted into spliced XBP1 (XBP1s), which then regulates folding and degradation genes. It can also activate the JNK pathway by binding to TRAF2-ASK1, mediating inflammation and cell death. ATF6 translocates to the Golgi apparatus, where it is activated by S1P and S2P cleavage and translocate to the nucleus. MFN2 maintains endoplasmic reticulum-mitochondrial coupling. Under conditions of severe stress, the UPR switches to pro-apoptotic pathways, thereby initiating inflammatory responses and pathways leading to cell death. In addition, ferroptosis and paraptosis may act in concert to further drive cell death.

ATF6 is canonically activated by dissociation from GRP78, followed by proteolytic cleavage by S1P and S2P and subsequent translocation to the nucleus [192, 199, 200]. Once in the nucleus, ATF6 induces the expression of XBP1 and molecular chaperone genes, thereby promoting cell survival. Although ATF6 is not a defined component of MAMs, its activity is modulated by the MAMs microenvironment through direct or indirect mechanisms [147, 201]. Notably, VAPB mutations or dysfunction have been shown to attenuate ATF6 activity [147, 201]. Collectively, these findings support a role for ATF6 in ER–mitochondrial crosstalk (Fig. 4).

### Mitochondrial functional homeostasis

Mitochondrial fusion promotes the integration of mitochondrial contents, repair of damaged mitochondria, and exchange of mtDNA [202]. MFN2, a GTPase that forms homo- or heterodimers, contributes to MAMs formation [203] and interacts with MAM-associated proteins such as the AMBRA1-ERLIN1 complex and PERK [204, 205]. By contrast, mitochondrial fission is a highly conserved process that is closely dependent on MAMs. Its core effector, Drp1, is recruited to ER-marked fission sites, where it oligomerizes into ring-like structures to drive mitochondrial membrane constriction and scission. This process requires the coordinated action of several OMM receptor proteins, including Fis1, MFF, and MiD49/MiD51, as well as ER-associated regulatory factors [206, 207]. The ER regulates mitochondrial fission through two major mechanisms. On the one hand, ER-associated proteins directly participate in the fission process. On the other hand, tubular ER physically wraps around mitochondria and induces initial constriction prior to Drp1 recruitment, thereby marking the site of fission. Subsequently, INF2-mediated F-actin polymerization cooperates with Drp1 to promote secondary fission [10, 208]. Furthermore, post-translational modifications of Drp1, such as phosphorylation and S-nitrosylation, influence not only its fission activity but also the stability of MAMs [209, 210]. B cell receptor-associated protein 31 (BCAP31), an ER membrane protein, interacts with Fis1 on the OMM, and its cleavage product p20BAP31 promotes Drp1 recruitment and mitochondrial fission. Additional proteins, including mitochondrial fission regulatory factor 2 (MTFR2) [211], brain-specific Ras-like protein 32 (Rab32) [212], FUNDC1 [213], STX17, and disulfide bond A oxidoreductase-like protein (DsbA-L) [214], also involved in Drp1 recruitment and the regulation of mitochondrial fission.

In COVID-19-related ARDS and LPS-induced ALI models, STING acts as a MAMs regulator that mediates Drp1 recruitment and its coordinated anchoring with the N-terminal fragment of GSDMD to the mitochondrial membrane through interactions with IP_3_R and STIM1, thereby linking mitochondrial fission to inflammatory responses. STING deficiency or treatment with the GSDMD inhibitor disulfiram (DSF) attenuates Drp1/GSDMD-dependent mitochondrial fission and reduces mtDNA release [147, 215, 216]. Moreover, the TNFα-induced IRE1α/XBP1s pathway promotes Drp1^S616^ phosphorylation and mitochondrial translocation by transcriptionally activating CDK1 and CDK5, thereby driving mitochondrial fission and dysfunction [217]. Notably, mtDNA replication within mitochondria has also been shown to be coordinated with fission events at the outer surface of the organelle, suggesting that in addition to ER–mitochondrial contact sites, intramitochondrial spatial determinants may contribute to fission-site positioning [218]. Taken together, whether MAMs constitute the central mechanism underlying mitochondrial fission-mediated lung injury remains an important question for future investigation.

Mitophagy primarily depends on the PINK1-Parkin signaling pathway. MAMs provides a structural platform for autophagosome formation, where the initiation factors such as ATG14L and ATG5 accumulate to drive autophagosome generation. The absence of MFN2 or PACS2 disrupts MAMs integrity and impairs autophagosome formation [145]. Moreover, ERS influences mitophagy through transcriptional regulation. Specifically, ERS upregulates the transcriptional repressor ATF3, which binds to the PINK1 promoter and suppresses its transcription, thereby causing mitochondrial dysfunction, ROS accumulation, and reduced cell viability [188]. Together, this finding suggests that the ER-mitochondrial axis regulates MQC both by preserving MAM integrity and by regulating the PINK1-Parkin pathway. In addition, SERCA-mediated cellular Ca^2+^ homeostasis also contributes to autophagy regulation, as SERCA deficiency impairs autophagosome-lysosome fusion and Rab7 recruitment, whereas SERCA activation promotes autophagosome-lysosome-like structures formation [219, 220].

In IPF patients, ERS impairs mitochondrial function in AECII by downregulating PINK1 [188, 221], a finding also confirmed in tunicamycin-treated mice [188]. Mechanistically, ERS triggers UPR and induces ATF3 expression. As a transcriptional repressor, ATF3 binds to the PINK1 promoter, leading to mitochondrial depolarization, elevated ROS levels, and reduced cell viability. Likewise, in SiO₂-induced chronic pneumonia, the PINK1-Parkin pathway regulates mitophagy to maintain mitochondrial homeostasis [158]. Moreover, the environmental toxin PFAAs induces ATF4 and p-IRE1 through ERS, thereby activating both mitophagy and ER-phagy. These protective effects depend on MAPK activation and PI3K/Akt inhibition. Consistently, inhibition of autophagy by Atg5 siRNA or chloroquine, but not by NAC, exacerbates PFAA toxicity, supporting a protective role for autophagy in response to organelle damage [222].

**Fig. 5:**
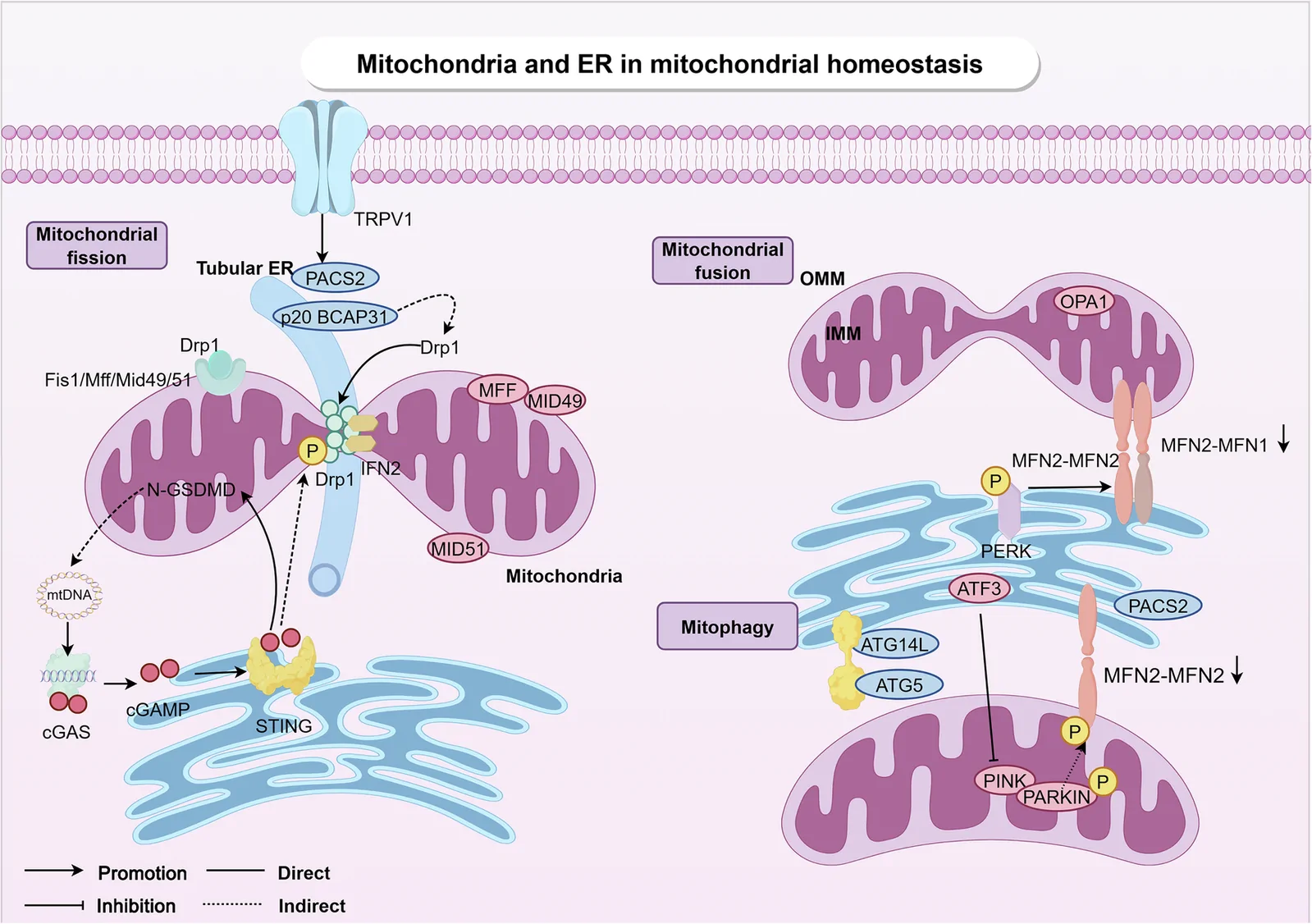
Roles of endoplasmic reticulum-mitochondria communication in mitochondrial homeostasis. Mitochondrial fission. Mitochondrial fission is driven by Drp1 in collaboration with INF2 in the endoplasmic reticulum to drive mitochondrial contraction and achieve secondary cleavage. BCAP31 is an ER membrane protein, and its cleavage product p20BAP31 increases during the transport of Ca²⁺ from ER to mitochondria, promoting Drp1 recruitment and driving mitochondrial fission. Drp1 and its adaptor proteins Fis/Mff/Mid49 and MiD51 are recruited to mitochondria. PACS2 is located at the ER-mitochondria interface, where it mediates BAP31 function and maintains ER-mitochondria contacts, supporting mitochondrial fission and mitophagy. The STING-N-GSDMD-mtDNA positive feedback loop can induce inflammation and cell pyroptosis, and STING deficiency weakens the synergistic effect of Drp1 and N-GSDMD after LPS stimulation. **Mitochondrial fusion:** Mitochondrial fusion is mediated by MFN1/MFN2 in the outer mitochondrial membrane, which in turn is mediated by OPA1 in the inner mitochondrial membrane. MFN2 also acts as a MAMs protein, participating in the establishment of the ER-mitochondria junction. **Autophagy:** In mitophagy, PINK1 phosphorylates MFN2 as a mitochondrial receptor for Parkin, leading to mitochondrial ubiquitination and autophagic clearance. ATG14L and ATG5 can relocalize to MAMs to initiate autophagy, while the loss of MFN2 or PACS2 disrupts MAMs structure and hinders autophagosome formation.

F-actin collaborates with focal adhesion proteins and myosin to form the contractile apparatus, which is regulated by PKC-MLCK-MLC phosphorylation. Meanwhile, the microtubule network provides essential support for intracellular dynamics and structural organization. Excessive ROS generation and elevated intracellular Ca²⁺ levels disrupt actin dynamics and cytoskeletal integrity, thereby contributing to smooth muscle contractile dysfunction. Antioxidant interventions and modulation of ER Ca²⁺ channels can partially restore cytoskeletal homeostasis and contractile function. In vitro human bronchial smooth muscle cell model, cigarette smoke extract (CSE) impaired actin dynamics and reduced the expression of key cytoskeletal proteins *via* the PKC-MLCK-MLC pathway, leading to abnormal contractile responses. Notably, mitoTEMPO partially restored bronchial contractility following CSE stimulation [167] (Fig. 5).

### Cell death

The evidence indicates that MAMs are broadly involved in multiple cellular processes, including Ca²⁺ transport, ROS signaling, lipid metabolism, ERS, and the maintenance of mitochondrial homeostasis. Dysregulation of these processes ultimately determines the activation of cell death programs. During apoptosis, MAMs facilitate Ca^2+^ transfer from the ER to mitochondria *via* the IP_3_R-VDAC-MCU complex, thereby triggering mPTP opening and initiating mitochondrial-mediated apoptosis [223]. In addition, anti-apoptotic Bcl-2 family members, including Bcl-2, Bcl-XL, and Mcl-1, bind to IP₃R or VDAC1 to suppress Ca²⁺ release [224, 225]. Conversely, pro-apoptotic members such as Bax and Bak promote ER Ca^2+^ efflux by displacing Bcl-2 from IP_3_R1 [127, 226]. PACS2 also plays a key role in MAM-mediated apoptosis signaling. Under conditions of severe ERS, Bid undergoes dephosphorylation and binds to PACS2, facilitating its translocation from ER to mitochondria. Subsequent cleavage by caspase-8 generates truncated Bid (tBid), which activates Bax/Bak and initiates apoptosis [227, 228]. Furthermore, the MAM-associated protein MFN2 interacts with ER membrane protein BAP31 to promote p20 BAP31 activation and the conversion of procaspase-8 into its active form. This process also enhances Ca²⁺ translocation to mitochondria, thereby establishing a positive-feedback loop in apoptosis [149, 150]. In addition, MAM-associated proteins such as SERCA2 can also induce apoptosis through Ca^2+^-dependent signaling. In LPS-induced ALI and IPF-related models, reduced expression of BAP31 or PACS2 is associated with increased apoptosis in ATII cell, whereas restoration of their expression alleviates ERS and mitochondrial injury, an effect that may be related to pirfenidone treatment [32, 147, 229].

In addition to Ca²⁺-dependent apoptosis, MAMs function as a key signaling platform that couples ERS to mitochondrial apoptosis. Sustained ERS activates the downstream transcription factors XBP-1 and CHOP *via* UPR transmembrane proteins, including IRE1α, PERK, and ATF6, thereby modulating pro-apoptotic Bcl-2 family proteins [230]. Mechanistically, IRE1α activates JNK signaling through recruitment of the tumor necrosis factor (TNF) receptor-associated factor 2 (TRAF2)-apoptosis signal-regulating kinase (ASK1) complex, promoting its translocation to mitochondria and inducing Bax/Bak activation, ultimately triggering caspase-dependent apoptosis [231–233]. Consistent with this mechanism, previous studies have shown that in lung injury, asbestos exposure or oxidative stress markedly upregulates IRE1, spliced XBP-1, and CHOP in alveolar epithelial cells from patients with IPF, accompanied by enhanced ER Ca²⁺ release and increased apoptosis [234, 235]. Similarly, in LPS-induced ALI/ARDS models, activation of the IRE1α-TRAF2-ASK1-JNK axis promotes Bim phosphorylation, Bax/Bak oligomerization, and caspase-dependent apoptosis, highlighting this pathway as a critical signaling hub integrating ER-mitochondrial apoptosis signaling during lung injury [197].

Ferroptosis is a regulated form of cell death driven by iron-dependent ROS accumulation and characterized by excessive lipid peroxidation [236–239]. MAMs contain several key enzymes involved in phospholipid biosynthesis and represent a principal site for the initiation of phospholipid peroxides formation. Accordingly, disruption of MAMs suppresses phospholipid peroxidation and ferroptosis, whereas stabilization of these contacts enhances phospholipid peroxidation [240]. In ALI, mtROS-mediated MFN2-PERK interaction is at least partly involved in arsenic-induced lipid peroxidation and ferroptosis through disruption of MAMs homeostasis [31].

Paraptosis is a novel form of regulated cell death characterized by protein synthesis-dependent cytoplasmic vacuolation, manifested as both large and small vacuoles, accompanied by ER and mitochondrial dilation, and is therefore considered a distinct, apoptosis-independent cell death pathway [31, 241]. In A549 cells, excessive ERS induces glutamine deprivation and trigger paraptosis, thereby promoting CHOP accumulation in mitochondria. This process disrupts MMP, enhances ROS generation, and impairs mitochondrial OXPHOS [230].

### Others

Current research suggests that mitochondria and the ER may function as important intracellular Mg²⁺ reservoirs, with matrix-free Mg²⁺ concentrations similar to those in the cytoplasm, indicating dynamic Mg²⁺ exchange between these organelles. However, unlike the Ca²⁺ transport through IP₃R-RyR-mediated ER–mitochondrial signaling, the transmembrane transport mechanism of Mg²⁺ at MAMs remains unclear. Mitochondrial transport proteins such as Mrs2 and SLC41A3 have been implicated in Mg²⁺ uptake and efflux, whereas the relevant ER channels remain unidentified [242, 243]. L-lactate has been reported to trigger dynamic Mg²⁺ exchange between the ER and mitochondria by stimulating Mg²⁺ release from the ER followed by mitochondrial uptake, with Mrs2 mediating mitochondrial Mg²⁺ accumulation [244]. Overall, these findings suggest that, although the molecular mechanisms of Mg²⁺-dependent ER–mitochondrial coordination remain incompletely understood, Mg²⁺ may constitute a transmembrane inter-organelle signaling network analogous to Ca²⁺ and contribute to the regulation of energy metabolism, gene expression, and cell development.

## Targeted therapy and clinical translation

In recent years, numerous studies have indicated that MAMs are not merely an “accompanying structure” of ERS or apoptosis, but rather central regulatory hubs that integrate Ca²⁺ transport, energy metabolism, and MQC in lung injury-related diseases such as ALI, ARDS, and pulmonary fibrosis. Their structural and functional disruption has clear pathogenic relevance. Accordingly, therapeutic strategies targeting MAMs differ from traditional broad-spectrum anti-inflammatory or anti-apoptotic interventions in that they aim to restore ER-mitochondria contacts, Ca²⁺ coupling, and precise inter-organelle signaling (Table 4).

**Table 4:** MAMs-based targeted therapy for lung diseases.

Compound	Target	Associated MAMs effect	Models	Ref.
Genipin	PI3k/Akt	Inhibited mitochondria-dependent apoptosis, ERS, and inflammation	LPS-induced ALI mice model	[249]
Melatonin, Pirfenidone	BAP31	Upregulation of BAP31 ameliorates ERS and mitochondrial dysfunction-mediated apoptosis	LPS-induced ALI mice; ATII cells model	[229, 245]
Capsaicin	PACS2-TRPV1	Restored the structure of MAMs and reduced mitochondrial damage and ERS by enhancing the interaction between PACS2 and TRPV1.	MLE-12 cells model	[32]
TUDCA, diosgenin	VAPB-PTPIP51	Repairing the MERC structure alleviates mitochondrial damage, reduces fibroblast activation and ERS.	LPS-induced ALI mice; ATII cells model	[126, 246]
Metformin and Tideglusib	AMPK	Reduces ERS and associated AEC apoptosis	In a murine model of lung injury by P. aeruginosa non-lethal infected lung injury in mice model	[250]
Dendrobine	FAM134B	Upregulation of FAM134B-mediated ER autophagy and MAMs reduction	LPS-induced sepsis mice; THP-1 cells model	[126]
MitoQ	mtROS	Alleviated mtROS-induced MAMs dysfunction	As-induced ALI mice; MLE-12 cells model	[31]
NecroX	ROS	Inhibited mitochondrial oxidative stress and ERS	Polyhexamethylene guanidine (PHMG) and BLM-induced lung injury in mice model	[138]

In current research, therapeutic strategies that directly target key structural proteins or complexes of MAMs have shown the highest specificity and disease relevance. Studies indicate that modulation of representative MAMs molecules, including BAP31, PACS2-TRPV1, and VAPB–PTPIP51 plays a pivotal role in maintaining ER-mitochondrial contacts, regulating Ca²⁺ transport, and controlling the threshold for mitochondria-mediated apoptosis in lung injury. Melatonin upregulates BAP31 expression *via* ERK pathway activation [245], whereas pirfenidone alleviates ERS and improves mitochondrial damage by restoring BAP31 levels in ATII cells [229]. Capsaicin polysaccharide ameliorates MAMs dysfunction by upregulating PACS2-TRPV1. In parallel, TUDCA, diosgenin, and dendrobine enhance VAPB-PTPIP51 interaction, restore MAM integrity to alleviate mitochondrial damage, and ultimately reduce fibroblast activation and ERS [32, 126, 246]. These findings support a therapeutic strategy focused on targeting key structural and functional components of MAMs.

Current evidence suggests that therapeutic strategies directly targeting ER-mitochondrial Ca²⁺ coupling may offer promising MAM-based interventions. NecroX, an indole-based antioxidant that preferentially localizes to mitochondria, effectively scavenges mtROS [32, 247, 248]. In lung epithelial cells, NecroX reversed PHMG-induced shortening of the ER–mitochondrial distance, restored ER-mitochondrial Ca^2+^ coupling, and partially alleviated ER oxidation and ERS [138]. Likewise, in a TNF-α-induced PAH cell model, cistanche phenylethanoid glycosides (CPGs) suppressed abnormal Ca²⁺ transport and reduced eIF2α, CHOP, and VDAC1 expression through regulation of the MFN2-IP_3_R3-SERCA axis, supporting their role in MAM-associated Ca^2+^ regulation. Given that ERS promotes excessive ROS production and activates the Ca²⁺/XO/ROS/mPTP pathway, dual inhibition of ROS and ERS may offer a potential strategy for protecting against lung injury. Compounds such as metformin, tideglusib and genipin [249] may confer protection by modulating MAM-related signaling through the AMPK or PI3K/AKT pathways, thereby improving energy metabolism and suppressing ERS. Their protective effects are blocked by the PI3K inhibitor LY294002 [250]. Further research is needed to determine whether and how it directly regulates MAMs to exert its protective effects against lung injury.

From a clinical translation perspective, a more feasible strategy is to precisely intervene at specific MAM-associated targets, rather than simply altering the extent of ER-mitochondrial contact. In this regard, light- or chemical-inducible reversible dimerization systems, cell type-specific delivery platforms such as AAV vectors or lung-targeting nanocarriers, and tunable proximity-labeling and functional manipulation tools may provide new strategies for precise MAMs regulation [251–253]. Furthermore, in the absence of MAMs-specific small molecules, repurposing existing drugs allows for a systematic evaluation of the effects of marketed drugs on MAMs structural parameters and function. The potential for repurposing drugs such as metformin, pirfenidone, and TUDCA may therefore serve as candidates for identifying functionally biased modulators while maintaining lung homeostasis.

Despite the considerable therapeutic potential of MAM-related targets, their clinical translation remains challenge. First, off-target effects and systemic safety concerns represent major barriers. MAM-associated proteins such as SERCA, IP_3_R, MCU, and VAPB-PTPIP51 are widely involved in Ca^2+^ signaling and energy metabolism in multiple tissues, including the myocardium and neurons system. As a result, systemic interventions may impair cardiovascular or neurological function, highlighting the need for lung-specific delivery strategies to reduce systemic toxicity [254, 255]. Second, therapeutic efficacy is strongly stage-dependent. At different disease stages, the same target may require drastically opposed intervention approaches. For instance, enhancing MCU activity may be beneficial during the insufficient-coupling phase of viral infection phase [256], but may exacerbate Ca²⁺ overload during the acute injury phase [141]. Likewise, although SERCA activators are protective in viral infection models, their use during the over-coupling phase may further increase mitochondrial Ca²⁺ loading by augmenting ER Ca²⁺ stores [257, 258]. Therefore, the therapeutic timing and dosage window require further clarification.

In summary, therapeutic strategies targeting BAP31, PACS2, VAPB-PTPIP51, the MFN2-IP_3_R-SERCA axis, and their regulatory networks have demonstrated potential in ameliorating lung injury through modulation of MAMs. Collectively, these findings highlight the MAMs axis as a promising therapeutic target and support the gradual transition of precision treatment strategies from mechanistic investigation to clinical translation.

## Discussion

Numerous studies have shown that mitochondrial and ER dysfunction are central to the development and progression of lung injury-related diseases including COPD, ALI, ARDS, and pulmonary fibrosis. Mitochondrial dysfunction impairs energy metabolism, promotes excessive ROS production, and disrupts Ca²⁺ homeostasis, whereas sustained ERS and UPR activation induce alveolar epithelial cell injury, epithelial-mesenchymal transition, and enhanced inflammatory and fibrotic responses. Importantly, mitochondria and the ER do not operate independently, but are structurally and functionally integrated through MAMs. The evidence summarized in this review highlights MAMs not only as physical contract sites between mitochondria and the ER, but also as central regulatory hub controlling Ca²⁺ homeostasis, lipid metabolism, UPR signaling, mitochondrial homeostasis, and cell fate. Their dysfunction may represent a common mechanistic basis underlying the diverse pathological manifestations of lung injury-related diseases. Therefore, targeting MAMs structure and function may represent a promising therapeutic strategy for lung injury and an important direction for future precision medicine.

Although recent studies have revealed the important role of MAMs in lung injury-related diseases, several challenges still limit our understanding of their spatiotemporal plasticity and pathological significance in lung tissue. First, the MAMs function is inherently bidirectional. Excessive ER-to-mitochondria Ca²⁺ transfer causes mitochondrial Ca²⁺ overload and ROS burst, whereas reduced Ca²⁺ flux hinders ATP production and delay tissue recovery. These effects appear to depend on the pathological stage [253]. For example, pathological overactivation of IP₃R induced by HTV leads to cytoplasmic Ca²⁺ overload and exacerbates cellular injury, whereas during physiological or reparative phases, IP₃R is essential for MAMs formation and Ca²⁺ transfer to mitochondria [253, 259]. This “double-edged sword” property complicates therapeutic strategies aimed at simply increasing or decreasing MAMs activity. Likewise, although drug-induced organelle crosslinking can enhance ER-mitochondrial contact and improve energy metabolism, it may also promote mPTP opening and pro-apoptotic signaling [260]. Second, limitations in experimental models and cell type specificity remain major challenges. The lung comprises highly heterogeneous cell populations, including alveolar epithelial cells, endothelial cells, macrophages, and fibroblasts, and both the abundance and functional roles of MAMs may vary considerably across these cell types [32, 126, 195]. Traditional two-dimensional quantification based on random fields of view may mask cellular heterogeneity and fail to capture cell type-specific changes in MAMs. Moreover, most current evidence comes from immortalized cell lines such as A549, whose metabolic profile, mitochondrial function, and MAM-associated stress responses differ substantially from those of primary alveolar epithelial cells, especially AECII cells, thereby limiting translational applicability [261]. Future research should therefore combine primary cells, cell type-specific genetic tools, and single-cell or spatial omics approaches to achieve cell type-specific validation of MAMs function. Third, there is still a lack of lung tissue-specific molecular markers for MAMs, as well as methods for in situ molecular capture. Currently, proteins commonly used as MAMs markers include IP₃R, VDAC1, Grp75, MFN2, PACS2, Sig-1R, and the VAPB–PTPIP51 complex. However, most of these proteins are not exclusive to MAMs, but are also independently present in the ER or mitochondria, where they perform diverse physiological functions. For example, MFN2 is not only a key regulator of mitochondrial fusion but has also been implicated in ER–mitochondrial tethering [262], and its broad functional involvement may easily lead to unintended effects. Therefore, the identification of specific MAMs biomarkers and the development of molecular tools that enable precise regulation are crucial for maintaining and restoring lung tissue homeostasis. In the future, the combination of CRISPR-based organelle labeling [263], lung organoid models [264], and cell type-specific proximity labeling techniques, such as Apex2/TurboID or its split variant split-TurboID [253], is expected to enable the construction of cell type-resolved MAMs probes at the in situ level.

However, current imaging technologies still do not allow precise analysis of the dynamic changes in MAMs structure and function in lung injury-related diseases. Compared with conventional two-dimensional imaging systems, techniques such as electron tomography [208, 265–267], serial tilt tomography [267], focused ion beam scanning electron microscopy [268], and soft X-ray tomography [269] have enabled whole-cell three-dimensional imaging of MAMs. Nevertheless, data acquisition and three-dimensional reconstruction using these methods remain highly time-consuming, labor-intensive, and low-throughput, which limits their systematic application in disease models. With advances in transmission electron microscopy, researchers have been able to quantify key structural parameters of MAMs, including the gap width between the OMM and the ER membrane, as well as the length of the contact interface [209]. In addition, immunogold labeling has made it possible to detect specific proteins localized at MAMs [270]. However, these techniques are primarily applicable to fixed samples and are therefore difficult to use for tracking dynamic processes in living systems. In recent years, stimulated emission depletion super-resolution imaging technology has achieved a radial resolution of less than 50 nm [271]. Stochastic optical reconstruction microscopy, with a theoretical resolution of approximately 20 nm, was also used to observe MAMs [272]. For example, the combination of super-resolution imaging and confocal microscopy has been used to describe the aggregation of the apoptosis inhibitor vMIA, which localizes to MAMs [272]. However, these methods still have limited ability to quantify the overall extent or three-dimensional architecture of the MAMs interface. Notably, emerging technologies such as cryo-electron tomography combined with three-dimensional reconstruction [273], and the combination of real-time super-resolution imaging of living cells with cryo-electron tomography in “cryo-correlated light and electron microscopy” [274, 275], can systematically analyze the spatiotemporal distribution and functional heterogeneity of MAM-associated proteins across different pathological stages while maintaining high spatial resolution. The latest research has developed an adaptive learning physics-assisted light-field microscope (Alpha-LFM), which is based on a physics-assisted deep learning framework and an adaptive tuning strategy. This system achieves sub-diffraction-limit spatial resolution of up to ~120 nm while maintaining high temporal resolution and low phototoxicity. It enables rapid and gentle three-dimensional super-resolution imaging of intracellular dynamics at millisecond timescales, showing considerable promise for studying MAMs structure and function, including Ca²⁺ transients [275]. In the future, integration of high-resolution three-dimensional microscopy with proteomics is expected that a “spatiotemporal map” of MAMs in the context of lung injury, thereby providing a basis for precise targeted intervention. In the field of molecular probes, the dual-targeting fluorescent probe BDSTI simultaneously labels mitochondria and the ER, enabling synchronous imaging of their interaction. This probe has been used in a BLM-induced mouse model of pulmonary fibrosis to visualize and analyze MAMs alterations both in vivo and in vitro [276]. However, the field still faces a severe shortage of highly specific molecular probes that selectively detect MAMs components without interfering with their natural functional state, making it particularly challenging to investigate MAMs function while preserving normal cellular homeostasis. In the future, the development of r reversible, non-invasive molecular probes with high temporal resolution, together with optogenetic and chemogenetic strategies for precise MAMs modulation, will help construct a spatiotemporal map of MAMs in lung injury and lay a crucial foundation for precise, targeted interventions.

Looking ahead, MAMs research urgently requires breakthroughs in several key areas. first, light-induced and chemically induced systems may enable rapid and reversible control of ER–mitochondrial contact strength. Compared with traditional genetic approaches, these strategies offer greater temporal and quantitative precision in regulating organelle contacts and signaling, thereby helping to identify the “therapeutic window” and allowing dynamic manipulation of highly plastic cellular processes. Second, combining cell-type-specific delivery, such as AAV-based vectors or conditional transgenic approaches, with local administration strategies may facilitate the development of more selective tools for precisely targeting pathways, such as Ca²⁺ transport, UPR signaling, and mito-phagy, while minimizing systemic side effects.

## Conclusion

Lung injury remains a major challenge in both basic and clinical research. Growing evidence indicates that mitochondria and ER play are central to the complex network of pathological changes underlying disease progression. Although the ultrastructure and composition of MAMs are still not fully defined, continued advances in research techniques and disease models are expected to clarify the dynamic structural remodeling of MAMs during lung injury and identify key tethering proteins as potential biomarkers of disease onset and progression. Meanwhile, translational approaches such as targeted delivery systems, drug repositioning, and controllable biological tools may allow precise modulation of MAMs structure and function, thereby accelerating the transition from mechanistic insight to clinical intervention and ultimately improving therapeutic precision and efficacy in lung injury.
